# Identification, characterization and functional analysis of *AGAMOUS* subfamily genes associated with floral organs and seed development in Marigold (*Tagetes erecta*)

**DOI:** 10.1186/s12870-020-02644-5

**Published:** 2020-09-23

**Authors:** Chunling Zhang, Ludan Wei, Wenjing Wang, Wenquan Qi, Zhe Cao, Hang Li, Manzhu Bao, Yanhong He

**Affiliations:** 1 Key Laboratory of Horticultural Plant Biology, Ministry of Education; College of Horticulture and Forestry Sciences, Huazhong Agricultural University, Shizishan Street No. 1, Wuhan, 430070 China; 2 Crop Development Centre/Department of Plant Sciences, University of Saskatchewan, S7N5A8, Saskatoon, Canada

**Keywords:** Marigold, Floral organs, MADS-box genes, *AGAMOUS* subfamily genes, Functional analysis

## Abstract

**Background:**

*AGAMOUS* (*AG*) subfamily genes regulate the floral organs initiation and development, fruit and seed development. At present, there has been insufficient study of the function of *AG* subfamily genes in Asteraceae. Marigold (*Tagetes erecta*) belongs to Asteraceae family whose unique inflorescence structure makes it an important research target for understanding floral organ development in plants.

**Results:**

Four *AG* subfamily genes of marigold were isolated and phylogenetically grouped into class C (*TeAG1* and *TeAG2*) and class D (*TeAGL11–1* and *TeAGL11–2*) genes. Expression profile analysis demonstrated that these four genes were highly expressed in reproductive organs of marigold. Subcellular localization analysis suggested that all these four proteins were located in the nucleus. Protein-protein interactions analysis indicated that class C proteins had a wider interaction manner than class D proteins. Function analysis of ectopic expression in *Arabidopsis thaliana* revealed that *TeAG1* displayed a C function specifying the stamen identity and carpel identity, and that *TeAGL11–1* exhibited a D function regulating seed development and petal development. In addition, overexpression of both *TeAG1* and *TeAGL11–1* leaded to curling rosette leaf and early flowering in *Arabidopsis thaliana*.

**Conclusions:**

This study provides an insight into molecular mechanism of *AG* subfamily genes in Asteraceae species and technical support for improvement of several floral traits.

## Background

Flowers are the reproductive organs of a plant, which are regarded as an important morphological innovation in plant evolution. The MADS-box transcription factors are key elements in floral organ identity [1, 2], fruit and seed development [3, 4], and leaf and root development as well [5, 6]. Based on the genetic studies, a well-known ABCDE model is introduced to explain genetic regulation in floral organ determination. In this model, class A and E genes determine the sepals; class A, B, and E genes specify the petals; class B, C, and E genes regulate the stamens fate; class C and E genes control the carpel formation; and class D and E genes direct the ovule development [7, 8]. The *AGAMOUS* (*AG*) subfamily genes belonging to MADS-box classes C/D are involved in the regulation of floral organ, floral meristem, and fruit development. Previous reports demonstrated that *AG* subfamily genes are most likely to be arose from several paraphyletic lineages with multiple whole-genome duplication events (WGDs) in flowering plants, leading to possible subfunctionalization [9–12]. The first WGDs probably result in the generation of *AG* (class C) lineage and *AGAMOUS-LIKE11*(*AGL11*, class D) lineage [10, 11]. The *AG* lineage then undergoes the second WGDs in core eudicots, resulting in two sub-clades of *euAG* and *PLENA (PLE)* [9, 10].

The genes in *AG* lineage determining the floral meristem development and reproductive organ (stamen and carpel) identity [13] were firstly identified in Arabidopsis (*Arabidopsis thaliana*, *AG*) [13] and Antirrhinum (*Antirrhinum majus*, *PLE*) [10]. In Arabidopsis, the class C genes prevent the class A genes from functioning in inner whorl floral organs, which is clearly supported by reducing the *AG* expression in Arabidopsis, resulting in homeotic mutation of reproductive organs such as petal-like stamens and sepal- or petal-like carpels [14–16]. The function of *AG* lineage genes has been previously characterized in other eudicot species including Chrysanthemum (*Chrysanthemum morifolium*) [17], Petunia (*Petunia hybrida*) [18], Populus (*Populus trichocarpa*) [2], and also in monocot species such as Rice (*Oryza sativa*) [19].

Then, the *FLORAL BINDING PROTEIN7* (*FBP7*) and *FLORAL BINDING PROTEIN11* (*FBP11*) regulating ovule identity in petunia were determined as class D genes, and these two genes belong to the *AGL11* lineage [20, 21]. In Arabidopsis, there are three D class genes, named *SEEDSTICK* (*STK*; formerly known as *AGL11*), *SHATTERPROOT1* (*SHP1*), and *SHATTERPROOT2* (*SHP2*), which redundantly overlap in their function in regulating ovule development [22–24]. This is verified by the phenotypes of single gene and triple gene mutants. In triple mutant, integuments are transformed into carpelloid structures, and female gametophyte development is interrupted just after megasporogenesis. However, in *stk* single mutant, only the ovule funiculus is larger than that of the wild type, and the mature seeds are not detached from the silique [24–27]. In addition, the similar phenotype changes have been reported in cultivated Grapevine (*Vitis vinifera)* [28] and Populus [2], indicating that *STK/AGL11* has a conservative function in regulating the ovule development.

Asteraceae is one of the largest plant families of flowering plants, which bears a unique head-like inflorescence consisting of hundreds of ray florets and disk florets. For head-like inflorescence, the outer are the sterile ray florets without stamen, and the inner are the fertile disk florets with complete four whorl organs. There are multiple floret morphological traits existing in a single inflorescence, such as fertility, symmetry, and organ fusion. Therefore, Asteraceae provides an unparalleled opportunity to study the genetic regulation of the above-mentioned phenomena. Previous studies have revealed that the auxin and genes *LFY* and *UFO* are involved in pattern formation of the head flower, and that *CYC*-like genes regulate the flower symmetry [29–31]. The MADS-box family genes play an important role in the regulation of floral meristem and floral organ development, and their functions in regulating the formation of head-like inflorescence in Asteraceae have been a research hotspot. Until now, the functions of class B genes have been reported in many Asteraceae species [32–34], but there have been few reports on *AGAMOUS* subfamily genes in Asteraceae species. Up to now, the *AG* gene functions in Gerbera (*Gerbera hybrid*) [35], Chrysanthemum [36], and Sunflower (*Helianthus annuus*) [37] have been reported, and the results reveal that *AG* function in Asteraceae is conservative in specifying the stamen and carpel identity. Unlike *AG* lineage genes, the functions of *AGL11* lineage genes have not been reported in Asteraceae.

Marigold (*Tagetes erecta*) is a popular ornamental plant. As a member of Asteraceae family, marigold has typical head flower consisting of two morphologically distinct types with ray (sterile) florets in the periphery and disk (fertile) florets in the center. The growth period of marigold is 2–3 months from sowing to flowering. In the evolutionary history of Asteraceae family, marigold undergoes a long evolutionary process, and it is located in a derived Calenduleae clade [38]. These characteristics make marigold an important plant in the study of floral organ development. Here, two *AG* lineage genes (*TeAG1* and *TeAG2*) and two *AGL11* lineage genes (*TeAGL11–1* and *TeAGL11–2*) of marigold were cloned, their expression patterns were investigated, and their subcellular localization was determined. Yeast two-hybrid assay and ectopic transformation were conducted to predict the gene functions in the formation of floret organs and the development of seeds.

## Results

### Isolation and molecular characterization of marigold *AGAMOUS* subfamily genes

**Fig. 1:**
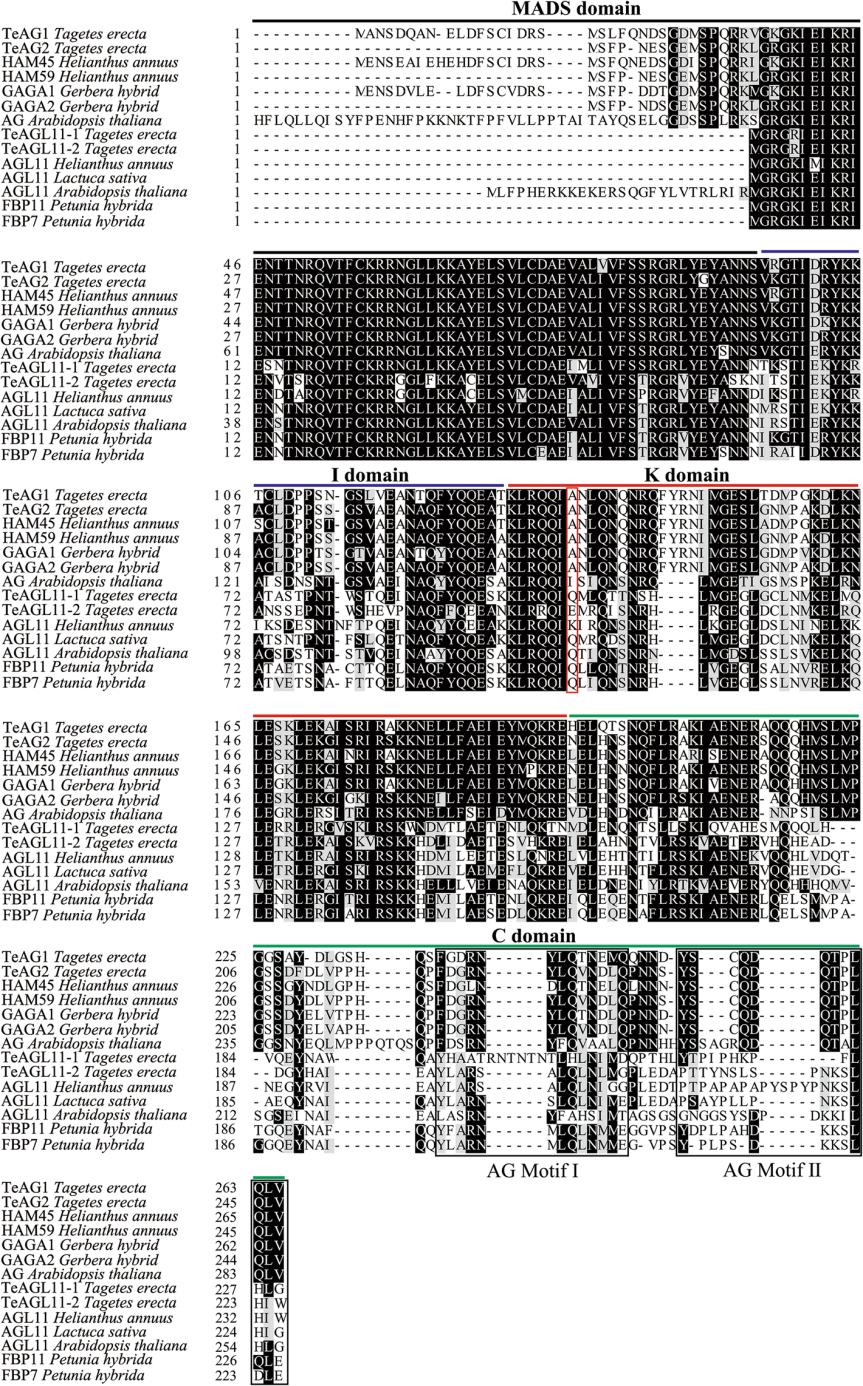
Multiple alignments of the predicted amino acid sequence of TeAG1, TeAG2, TeAGL11–1 and TeAGL11–2. The MADS domain is marked with black line. The I domain is marked with  blue line. The C domain is marked with green line. The K domain is marked with red line. The red box in K-domain indicates the conservative amino acid residues. The black boxes in the C-domain indicate the AG motif I and motif II, respectively

**Fig. 2:**
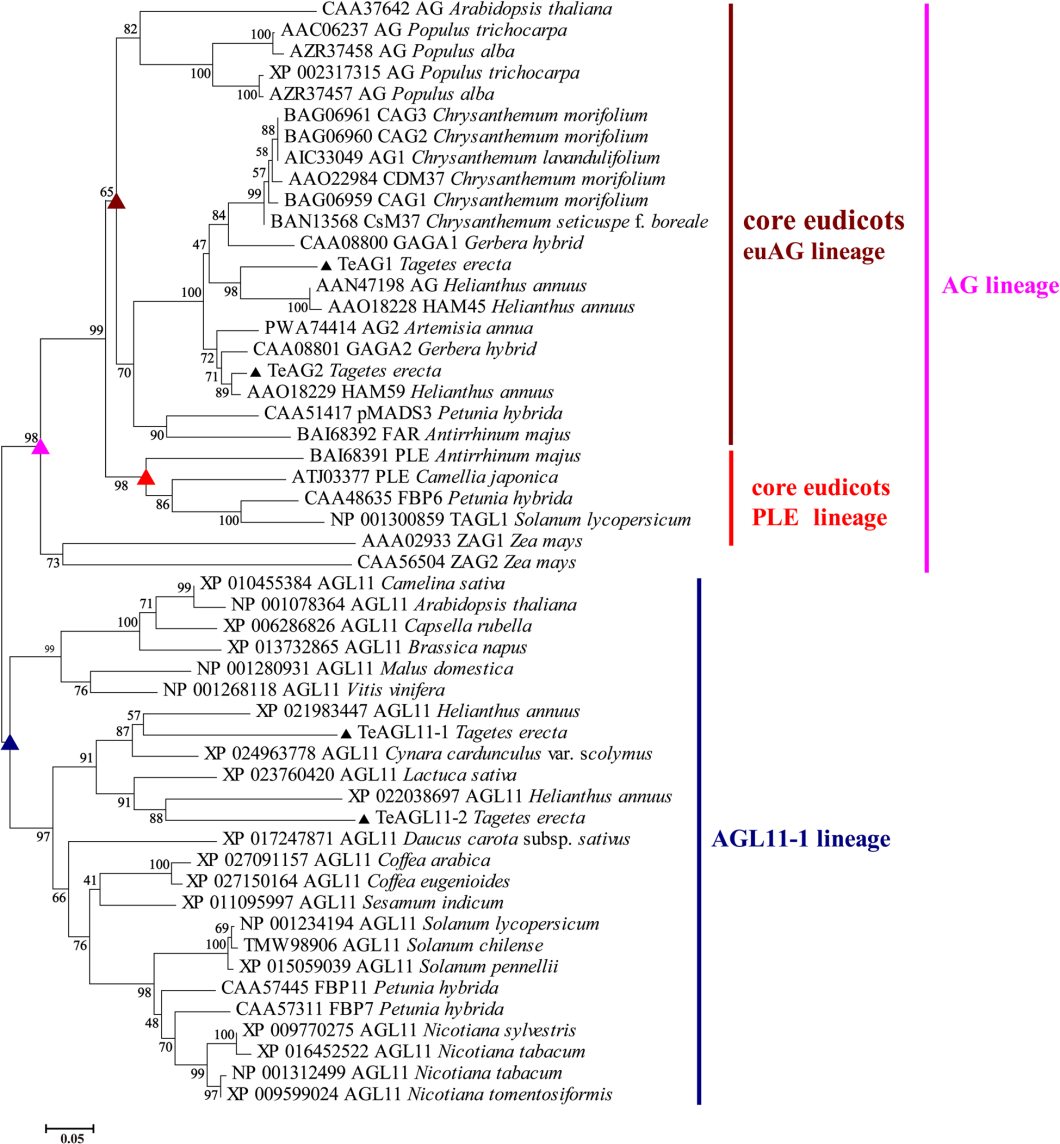
Phylogenetic tree based on the amino-acid alignment of AG and AGL11 proteins. The tree was generated with the MEGA v6.0 software, using the neighbor-joining (NJ) method and 1000 bootstrap replicates. The TeAG1, TeAG2, TeAGL11–1 and TeAGL11–2 are marked with black stars

To study the functions of *AG* subfamily genes in marigold*,* we amplified *TeAG1* (991 bp), *TeAG2* (837 bp), *TeAGL11–1* (735 bp), and *TeAGL11–2* (831 bp). Their sequences included the open reading fragment, partial 5′ untranslated region and partial 3′ untranslated region. Multiple alignment with other typical C/D proteins from model plants and Asteraceae species showed that these four proteins were typical MADS-box proteins containing MADS-domain, I-domain, K-domain with conservative amino acid residues, and AG motif I and AG motif II in C-terminal end (Fig. 1). Phylogenetic analysis using neighbor-joining (NJ) method showed that these proteins were divided into two main branches of AG and AGL11 lineages corresponding to the MADS-box class C and class D genes, respectively (Fig. 2). The first WGDs was found to have occurred during evolutionary history of marigold. Two C class proteins TeAG1 and TeAG2 were clustered to core eudicot euAG lineage, and two D class proteins TeAGL11–1 and TeAGL11–2 were clustered into AGL11 lineage (Fig. 2). TeAG1 and TeAG2 proteins are putative orthologs of Sunflower HAM45 and HAM59 proteins, respectively, both of which shared amino acid identity as high as over 85% (Table S2). TeAGL11–1 and TeAGL11–2 proteins shared high similarity with their orthologs Sunflower*s* HaAGL11–1 and HaAGL11–2 proteins with amino acid identity of 70.76 and 65.38%, respectively (Table S3).

### Expression patterns of *TeAG* and *TeAGL11* genes

**Fig. 3:**
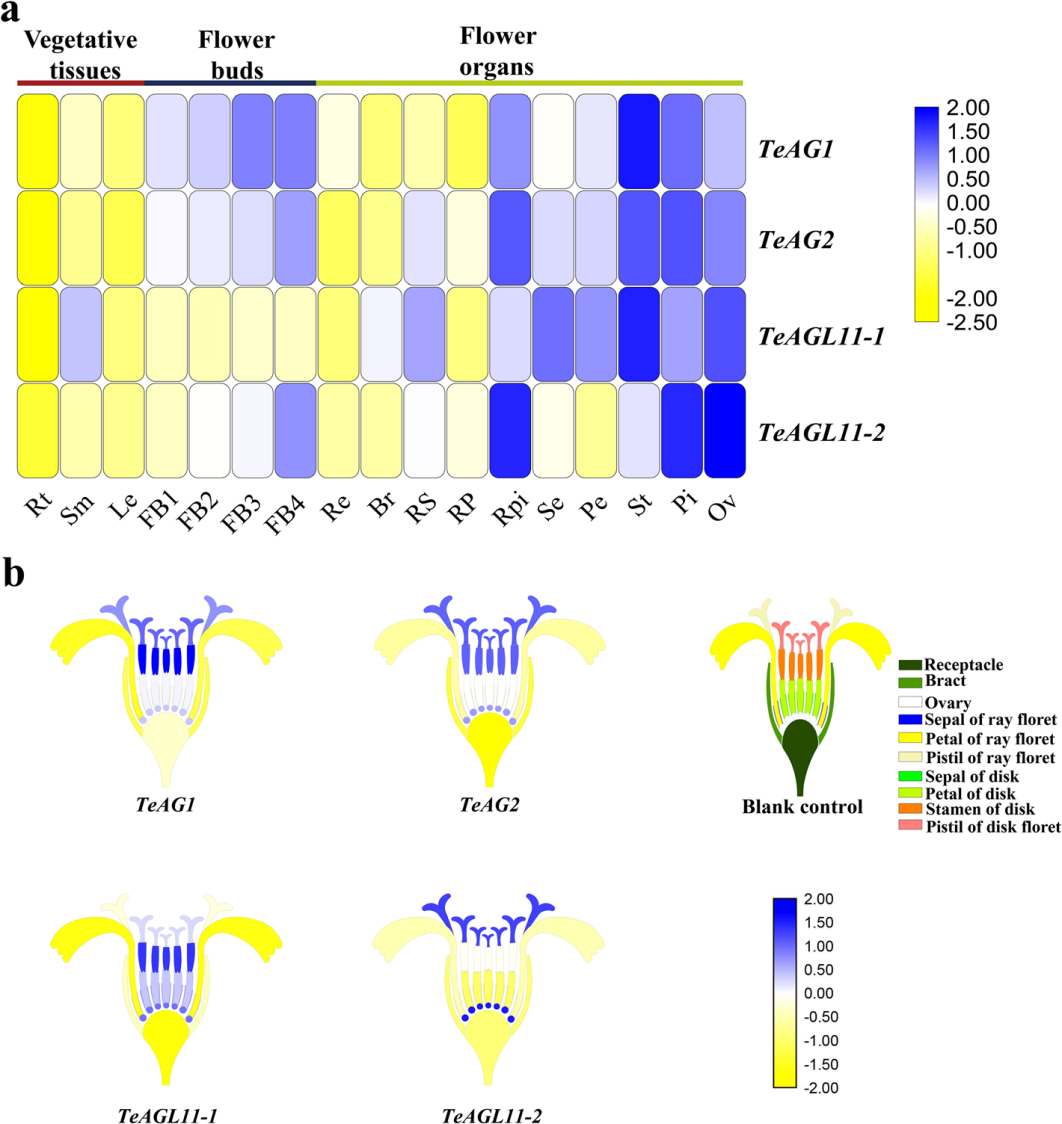
Expression levels of *TeAG* and *TeAGL11* genes in different tissues and organs of marigold. (**a**) Heatmap of relative expression of *TeAG1, TeAG2, TeAGL11–1* and *TeAGL11–2* genes by qRT-PCR in different tissues and organs. Rt: root; Sm: stems; Le: leaves; FB1-FB4: flower buds were 0-1 mm, 2-3 mm, 4–5 mm and 6-7 mm in diameter, respectively; Re: receptacle; Br: bract; RS: sepal of ray floret; RP: petal of ray floret; RPi: pistil of ray floret; Se: sepal of disk floret; Pe: petal of disk floret; St: stamen of disk floret; Pi: pistil of disk floret; Ov: ovary. (**b**) Heatmap of *TeAG1, TeAG2, TeAGL11–1* and *TeAGL11–2* genes in the inflorescence of marigold based on the relative expression by qRT-PCR. Blank control: structural model of capitulum in *T. erecta*, different colors represent different floral organs

Here, qRT-PCR was conducted to investigate the expression patterns of the four genes in marigold. In order to determine whether genes’ transcripts were stage-dependent or not, we preliminarily detected their expression levels at four stages of floral buds (FB1-FB4). The qRT-PCR analysis showed that the transcript levels of *TeAG1*, *TeAG2,* and *TeAGL11–2* showed an increase tendency during floral bud development, while the expression level of *TeAGL11–1* was very weak and exhibited no significant changes in four floral bud development stages (Fig. 3a, Fig. S1, Table S6).

We further analyzed the expression levels of the four genes in vegetative tissues, and anthesis stage of flower organs (Fig. 3a, b, Fig. S1, Table S6). The results showed that these genes were highly expressed in floral organs. The *TeAG1* and *TeAG2* were more preferentially expressed in reproductive organs (stamens, pistils and ovaries) than in sepals and petals. Interestingly, the transcript level of *TeAG1* was significantly higher in stamens than in pistils and ovaries, while that of *TeAG2* was higher in stamens and pistils than in ovaries. The expression patterns of the two *AGL11* genes varied in floral organs. *TeAGL11–1* had a wide expression region in disk florets, including sepals, petals, stamens, and pistils, whereas this gene was detected only in sepals and pistils of ray flowers, as well as in ovaries. Remarkably, the high expression level of *TeAGL11–1* was detected in stamens. In contrast, the *TeAGL11–2* was higher expressed in pistils, and ovaries than in stamens, sepals, and petals.

### Subcellular localization of TeAG and TeAGL11 proteins

**Fig. 4:**
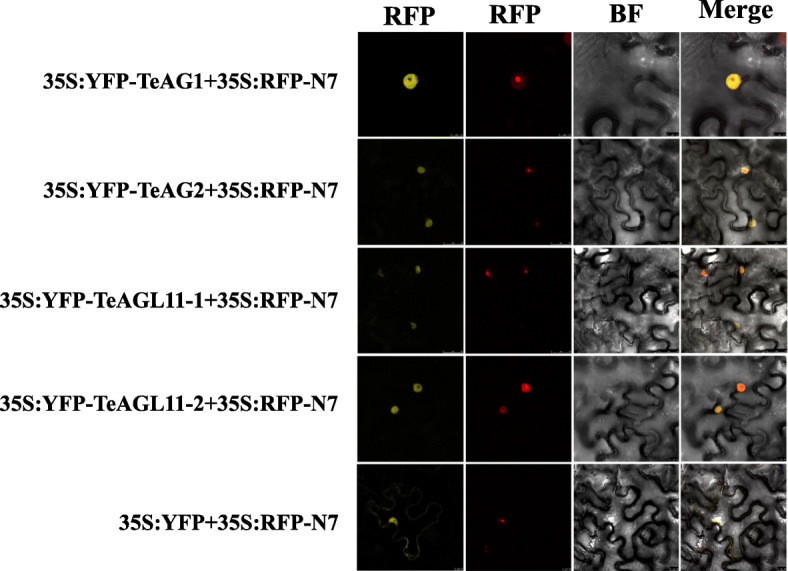
Subcellular localization of the AGAMOUS-like subfamily proteins of marigold*.* These four fusion proteins were driven by *35S* promoter and transiently expressed in tobacco leaf. Photographs were obtained with a confocal microscope. *35S:YFP* as a negative control; *35S:RFP-N7* as a nucleus controls. YFP: yellow fluorescence; REP: red fluorescence; BF: bright field image; Merge: merged images of Bright, YFP and REP fields

To gain an insight to the subcellular localization of these four genes, four fusion vectors *35S:YFP-TeAG1*, *35S:YFP-TeAG2*, *35S:YFP-TeAGL11–1*, and *35S:YFP-TeAGL11–2* were transiently co-transformed with *35S:RFP*-N7 vector into the leaf of tobacco*,* respectively. The fluorescence signals of these four fusion vectors were mainly observed in the nucleus outside the nucleolus (Fig. 4).

### Protein interactions of TeAG and TeAGL11

To confirm the interaction among the four proteins, the yeast two-hybrid experiment was performed. Self-activation of BD constructs was assessed. The results indicated that no autoactivation was observed (Fig. S2a). Although TeAG1 and TeAG2 proteins shared a high similarity in sequences, their interaction manner with other AGAMOUS subfamily proteins were different. As shown in Table 1 and Fig. S2b, the TeAG2 formed heterodimers with TeAG1, TeAGL11–1, and TeAGL11–2, and formed homodimer with itself. However, the TeAG1 only interacted with TeAG2 and TeAGL11–1, but it formed no homodimer. The TeAGL11–1 and TeAGL11–2 showed a limited interactive ability with other AGAMOUS subfamily proteins. Neither homodimer nor heterodimer were formed through the interaction between AGL11–1 and AGL11–2. TeAGL11–1 and TeAGL11–2 interacted with TeAG2 unidirectionally. In addition, TeAGL11–1 strongly interacted with TeAG1, while TeAGL11–2 had no ability to interact with TeAG1.

**Table 1:** Interactions of marigold TeAG and TeAGL11 proteins detected by yeast two-hybrid assays

	AD-TeAG1	AD-TeAG2	AD-TeAGL11–1	AD-TeAGL11–2	AD-empty	AD-T7
BD-TeAG1	–	++	++	–	–	/
BD-TeAG2	++	++	+	++	–	/
BD-TeAGL11–1	++	–	–	–	–	/
BD-TeAGL11–2	–	–	–	–	–	/
BD-empty	–	–	–	–	–	/
BD-53	/	/	/	/	/	/

Note: ++, strong interaction; +, weak interaction; −, no interaction, / not determined

### Dramatic effect of overexpression of *TeAG1* in Arabidopsis on sepal and petal identity

**Fig. 5:**
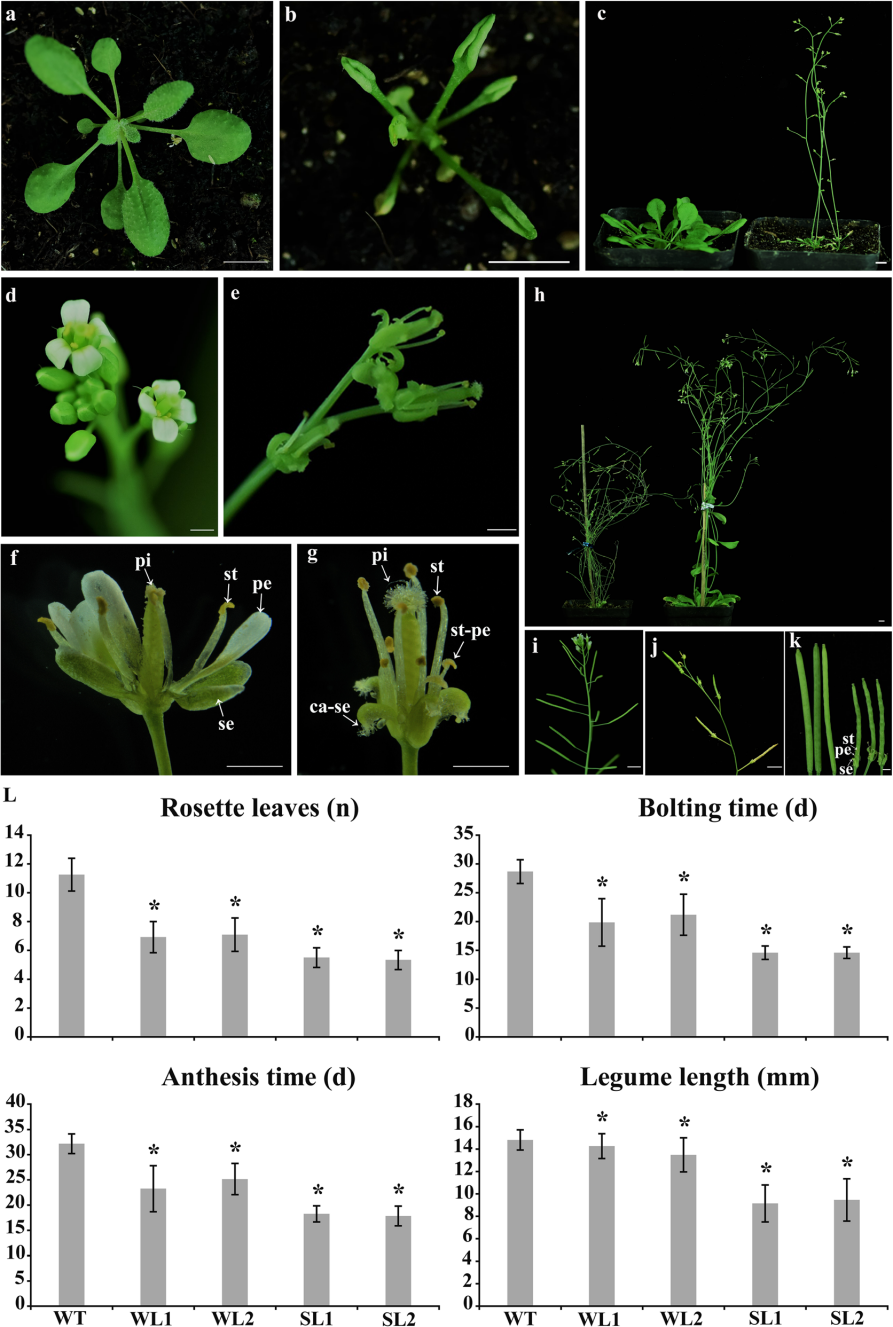
Abnormal morphology of transgenic Arabidopsis plants of constitutively expressed *TeAG1* gene*.* **a**-**k** The morphological trait of *Sl-TeAG1* lines and wild-type lines. (**a**) The wild-type seedling; (**b**) The transgenic seedlings with severe curled rosette leaves; (**c**) Wild-type (left) and early flowering transgenic plant (right); (**d**, **f**) normal flowers of wild-type; (**e**, **g**) mutant flowers of the transgenic plant; (**h**) Wild-type (right) and the dwarfing transgenic plant (left); (**j**, **k**) The siliques of transgenic lines are short and yellowish-green with persistented sepals, petals and stamens compared to those from wild-type (**i**). se: sepal; pe: petal; st: stamen; pi: pistil, ca-se: carpelloid sepals; st-pe: stamen-like petal; WT1: wild-type line 1; WT2: wild-type line 2; WL1: *Wl-TeAG1* line 1; WL2: *Wl-TeAG1* line 2; SL1: *Sl-TeAG1* line 1; SL2: *Sl-TeAGL1* line 2. a-c, bar = 5 mm, d-k, bar = 1 mm. (**l**) Statistics for main morphological traits of the control and transgenic plants, * significant difference at *P* < 0.05

To further study the functions of *TeAG1* and *TeAG2*, functional analyses were performed using ectopic expression in Arabidopsis. Eighteen *35S:TeAG1* transgenic lines and twenty-three *35S:TeAG2* transgenic lines were obtained. Transcript levels of *TeAG1* and *TeAG2* were further analyzed by semi-quantitative RT-PCR with the flower cDNA as templates (Fig. S3a, b). The *35S*:*TeAG2* transgenic lines did not show any evident morphological changes, compared with the wild type. However, five of the *35S*:*TeAG1* transgenic lines displayed severe phenotypes (named *Sl-TeAG1)*, seven showed weak phenotypes (named *Wl-TeAG1)*, and six had no remarkable phenotypic changes. Compared with the wild type, *Sl-TeAG1* and *Wl-TeAG1* transgenic lines displayed early flowering, rosette leaf curling, and small plant size (Fig. 5a, b, c, h, l, Table 2). Furthermore, only in *Sl-TeAG1* transgenic lines, normal sepal and petal formations were disrupted (Fig. 5d, e, f, g, Table 2). Homeotic conversion of sepal to pistil-like structure was detected at the top margin of sepals. Similar conversion of petal to stamenoid structure was observed (Fig. 5e, g, Table 2). The sepals, petals, and stamens retained at base of siliques (Fig. 5j, k). The siliques were more bumpy and smaller, and seed setting rate was lower than those of normal siliques in wild-type lines (Fig. 5, Table 2, S4).

**Table 2:** Mutant morphological traits of the transgenic plants via overexpression *TeAG1* and *TeAGL11–1*

	Rosette leaf	Flowering time	Sepal	Petal	Stamen	Pistil	Silique
*Sl-TeAG1*	Less and curled rosette leaves	Early flowerin	Carpelloid sepals	Stamen-like petals	–	–	Bumpy and small, low seed setting rate
*Wl-TeAG1*	Less and curled rosette leaves	Early flowerin	–	–	–	–	–
*Sl-TeAGL11–1*	Less and curled rosette leaves	Early flowering	Curled petal	–	–	–	Bumpy and small, almost seedless
*Wl-TeAGL11–1*	Less and curled rosette leaves	Early flowering	Curled petal	–	–	–	Bumpy and small, low seed setting rate

Note: -, no morphological change compared with wild-type lines

**Fig. 6:**
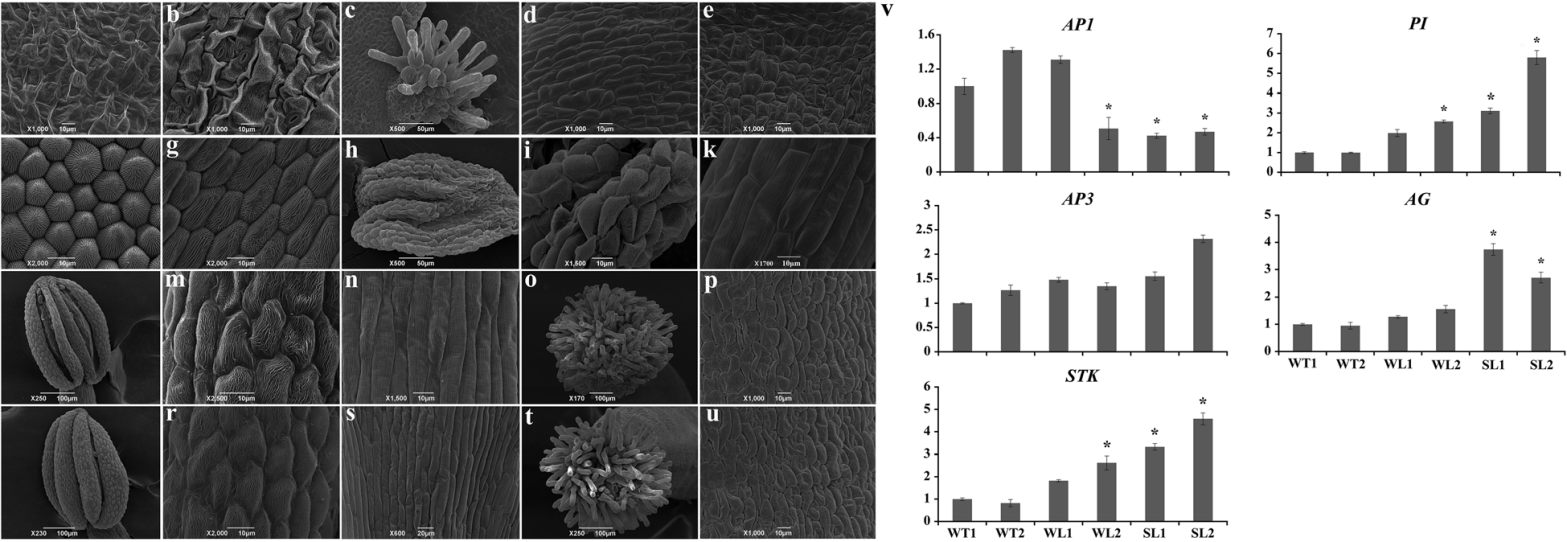
Scanning electron micrograph of floral organs and expression levels of genes related to floral organ development and seed formation between *TeAG1* transgenic Arabidopsis and wild-type lines. (**a**-**u**) Scanning electron micrograph of floral organs between *Sl-TeAG1* transgenic Arabidopsis and wild-type lines; (**a**, **b**) Adaxial (**a**) and abaxial (**b**) epidermis cells of sepals of wild-type; (**c**) The papilla-like cells at the top of the carpelloid sepals of transgenic plant; (**d**, **e**) Adaxial (**d**) and abaxial (**e**) epidermis cells of carpelloid sepals of transgenic plant; (**f**, **g**) The epidermal cells at the upper (**f**) and bottom portion (**g**) of the petals of wild-type; (**h**) The petals transformed into anther-like structure in transgenic lines; (**i**, **k**) The epidermal cells at the top (**i**) and bottom (**k**) part of anther-like structure; (**l**) The anther structure of wild-type lines; (**m**, **n**) The epidermal cells of anther (**m**) and filament (**n**) in wild-type lines; (**o**) The papilla cells of stigma in wild-type lines; (**p**) the epidermal cells of style in wild-type lines; (**q**) The anther structure of transgenic plant; (**r**, **s**) The epidermal cells of anther (**r**) and filament (**s**) in transgenic plant; (**t**) The papilla cells of stigma in transgenic plant; (**u**) The epidermal cells of style in transgenic plant. (**v**) Expression levels of genes related to floral organ development and seed formation in control and transgenic Arabidopsis flowers by qRT- PCR analysis. WT1: wild-type line 1; WT2: wild-type line 2; WL1: *Wl-TeAG1* line 1; WL2: *Wl-TeAG1* line 2; SL1: *Sl-TeAG1* line 1; SL2: *Sl-TeAGL1* line 2. * expression level of endogenous genes in transgenic plants was 2 times higher or 1/2 lower than that in wild-type plants

The four whorls of floral organs from *Sl-TeAG1* lines and wild-type lines were observed by SEM (i, j, k, l. 6). Compared with the sepals structure of wild type (Fig. 6a, b), a cluster of papilla-like cells occurred at the top of carpelloid sepals in transgenic lines (Fig. 6c), and the rough cells with stomata in normal sepals (Fig. 6a) were replaced by the smooth rectangle epidermis cells in adaxial surface of carpelloid sepals (Fig. 6d). In addition, the abaxial epidermis cells were converted from the normal rough types with stomata (Fig. 6b) into irregular smooth convex structure (Fig. 6e), which was similar to the epidermal cell structure of style (Fig. 6p). The second whorl of floral organs in the transgenic plants were converted into stamen-like petals with an anther-like structure (Fig. 6h) consisting of squamous cells (Fig. 6i). Furthermore, the epidermal cells in the lower region of the stamen-like petals were changed from a rough spindle structure (Fig. 6g) to a smooth filament-like structure (Fig. 6k, n). No obvious change was found in stamens and carpels in transgenic lines (Fig. 6l-u). In general, the overexpression of *TeAG1* in Arabidopsis resulted in homeotic mutation of flower organs such as carpelloid sepals and stamen-like petals.

Since the phenotypes of ectopic expression of *TeAG1* were visually focused on sepal and petal identity, the *AP1*, *AP3*, *PI*, *AG,* and *STK* genes in Arabidopsis were selected to detect whether their transcriptional levels were changed, based on the ABCDE model. The results (Fig. 6v, Table S7) showed that the transcript levels of *PI*, *AG* and *STK* were significantly up-regulated in transgenic line *Sl-TeAG1*, while that of *AP1* was remarkably down-regulated, suggesting that ectopic expression of *TeAG1* (class C gene) might suppress the expression levels of *AP1* (class A gene) in Arabidopsis. No significant difference in the transcript level of *AP3* (B class gene) was observed between wild type and *Sl-TeAG1* lines (Fig. 6v, Table S7). Compared with the results observed in *Sl-AG1* line, similar change tendency and mild expression level changes of *AP1*, *PI*, *AP3*, *AG* and *STK* were detected in *Wl-AG1* lines (Fig. 6v, Table S7).

### Effect of ectopic expression *of TeAGL11–1* in Arabidopsis on petals and seed development

**Fig. 7:**
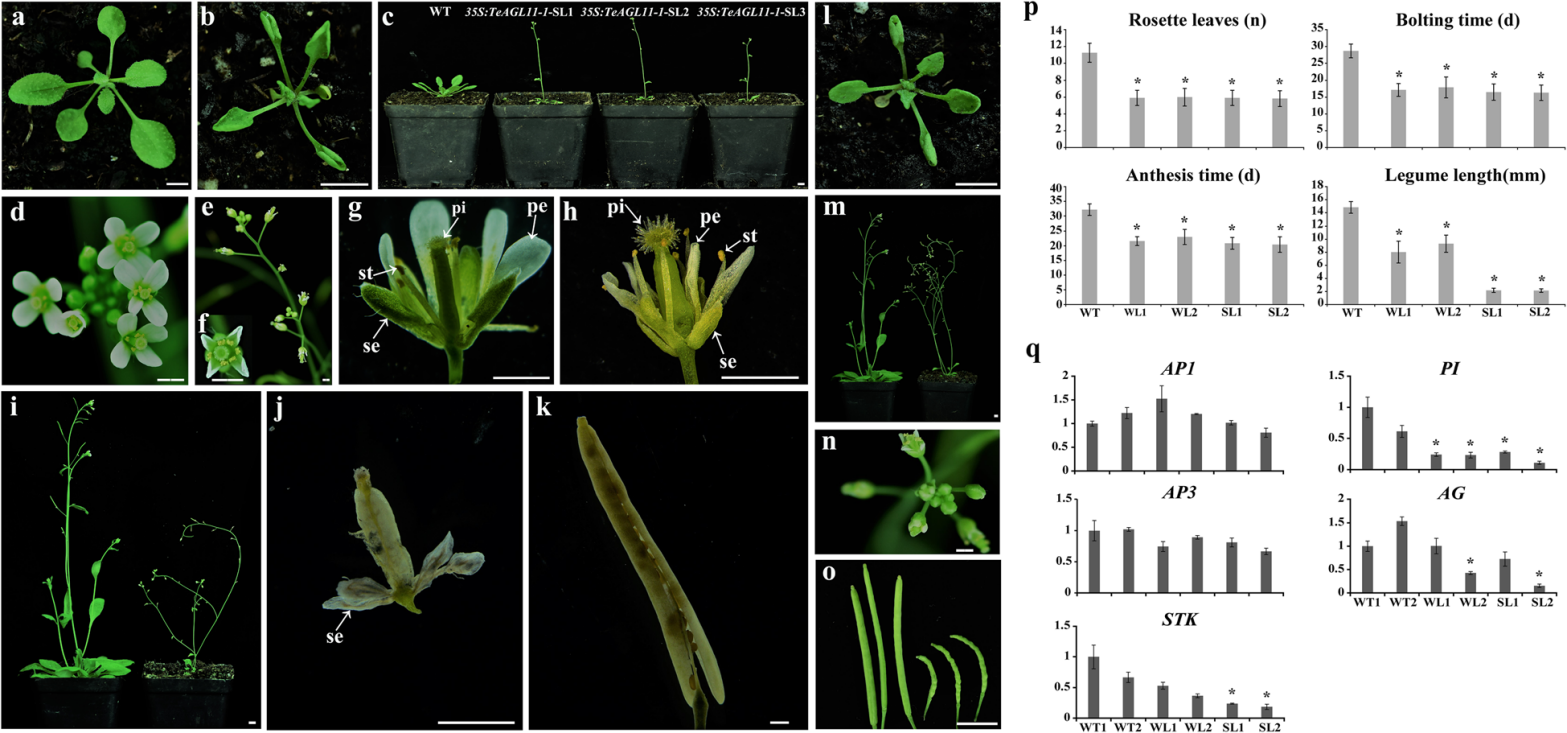
Abnormal morphology of transgenic Arabidopsis plants containing *35S:TeAGL11–1* and expression levels of genes related to floral organ development and seed formation. (**a**) The wild-type seedling. (**b**, **c**, **e**, **f**, **h**, **i**, **j**) Phenotype of *Sl-TeAGL11–1* lines. (**b**) The transgenic seedlings with severely curled rosette leaves. (**c**) Wild-type and *35S:TeAGL11–1* transgenic plant with early flowering. (**d**, **g**) Wild-type flowers. (**e**, **f**, **h**) Transgenic flowers. (**i**) Wild-type (left) and transgenic plant (right) with smaller plants. (**j**, **k**) The siliques are almost seedless and short with unabscised sepals (**j**), compared to those from wild-type (**k**). (**l**-**o**) Phenotype of *Wl-TeAGL11–1* lines. (**l**) The transgenic seedlings with severely curled rosette leaves. (**m**) Wild-type (left) and transgenic plant (right) with smaller plants. (**n**) Transgenic flowers. (**o**) The smaller siliques (right). se: sepal; pe: petal; st: stamen; pi: pistil, a-c, i, l, m. o, bar = 5 mm; d-h, j, k, n bar = 1 mm. (**p**) Statistics for main morphological traits of the control and transgenic plants, * significant difference at *P* < 0.05. (**q**) Expression levels of genes related to floral organ development and seed formation in control and transgenic Arabidopsis flowers by qRT- PCR analysis. WT1: wild-type line 1; WT2: wild-type line 2; WL1: *Wl-TeAGL11–1* line 1; WL2: *Wl-TeAGL11–1* line 2; SL1: *Sl-TeAGL11–1* line 1; SL2: *Sl-TeAGL11–1* line 2. * expression level of endogenous genes in transgenic plants was 2 times higher or 1/2 lower than that in wild-type plants

In order to investigate the function of *TeAGL11–1* and *TeAGL11–2*, the two genes were also ectopically expressed in Arabidopsis. We obtained twenty-one *35S:TeAGL11–1* transgenic lines with seven severe phenotype lines (*Sl-TeAGL11–1)*, ten weak phenotype lines (*Wl-TeAGL11–1)*, and four lines without phenotypic changes. We obtained forty-six *35S:TeAGL11–2* transgenic lines without any evident phenotypic alteration, compared with the wild-type lines. Transcript levels of *TeAGL11–1* and *TeAGL11–2* were further analyzed by semi-quantitative RT-PCR with flower cDNA as templates (Fig. S3c, d). The overexpression of *TeAGL11–1* in Arabidopsis resulted in upward and inward curling of rosette leaves, obvious petal curling, early flowering, and small plant size (Fig. 7a, b, c, d, e, f, g, h, i, l, m, n, p, Table 2). In *Sl-TeAGL11–1* lines, the siliques were almost seedless and smaller than those in wild-type lines, and the sepals were not detached from siliques (Fig. 7j, k, p, Table 2, S5). However, in *Wl-TeAGL11–1* lines, only bumpy and small siliques were observed (Fig. 7j, k, o, p, Table 2).

To explore whether the phenotype was affected by the expression of the endogenous gene *AP1*, *PI*, *AP3*, *AG,* and *STK* regulating the floral organs and ovule development, the qRT-PCR analysis was performed in the two severe phenotype lines, two weak phenotype lines, and two wild-type lines. As shown in Fig. 7q and Table S8, the transcript levels of *AP1* and *AP3* exhibited no significant difference among the six samples. The expression level of *PI* was obviously down-regulated in both *Sl-TeAGL11–1*and *Wl-TeAGl11–1* lines, but the expression level of *STK* was lower in *Sl-TeAGL11–1* lines than in *Wl-TeAGl11–1* lines, suggesting that the seedless phenotype in *Sl-TeAGL11–1* lines might be related to the downregulation of *STK* .

### Expression profile analysis of endogenous genes related to early flowering and curled leaves

**Fig. 8:**
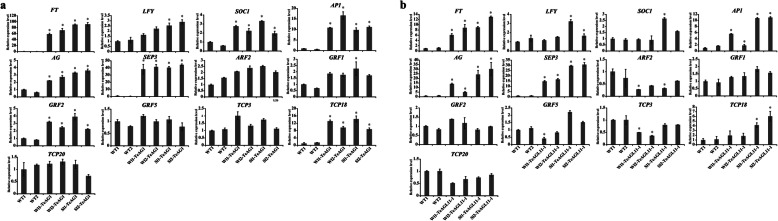
qRT-PCR analysis of endogenous flowering and leaf development-related genes in 10-day-old seedlings of the wild-type, *35S:TeAG1* and *35S:TeAGL11–1* transgenic lines of Arabidopsis. (**a**) qRT-PCR analysis of endogenous flowering and leaf development-related genes in *35S:TeAG1* transgenic lines. (**b**) qRT-PCR analysis of endogenous flowering and leaf development-related genes in *35S:TeAGL11–1* transgenic lines. * expression level of endogenous genes in transgenic plants was 2 times higher or 1/2 lower than that in wild-type plants

We also detected the expression level of endogenous genes related to flowering time (*AP1*, *FT*, *LFY*, *SOC1*, *AG* and *SEP3*) and curled leaves (*GRF1*, *GRF2*, *GRF5*, *TCP3*, *TCP18*, *TCP20*, and ARF2), when the transgenic and wild-type seedlings were 10 days old. As shown in Fig. 8, the expression levels of *AP1*, *FT*, *SOC1*, *AG* and *SEP3* were significantly higher in all the *35S:TeAG1* transgenic seedlings than in wild-type seedings. However, the expression level of the *LFY* was remarkably increased in *Sl-AG1* lines, and slightly increased in *Wl-AG1* lines (Fig. 8a, Table S9). Transcripts analysis of leaf development-related genes in *35S*:*TeAG1* transgenic seedlings indicated that expression levels of *ARF2*, *GRF1*, *GRF5*, *TCP20* and *TCP3* had no significant difference among the six samples (Fig. 8a, Table S9), whereas the expression levels of *GRF2* and *TCP18* were obviously higher than those in wild-type lines, suggesting that high expression of *GRF2* and *TCP18* might have caused the leaf curling.

In *Wl-TeAGL11–1* lines and *Sl-TeAGL11–1* lines, *AP1*, *AG*, *FT* and *SEP3* were strongly up-regulated. The expression levels of *SOC1* were increased in *Sl-TeAGL11–1* lines, and no significant difference in the expression level of *SOC1* was observed between *Wl-TeAGL11–1* lines and wild-type lines (Fig. 8b, Table S10). The results suggested that *AP1*, *AG*, *FT* and *SEP3* might contribute to the early flowering in *35S:TeAG11–1* transgenic lines. Expression levels of leaf development-related genes showed a complex expression pattern in *35S:TeAGL11–1* transgenic lines. The transcript level of *TCP18* in *Sl-TeAGL11–1* lines was up-regulated and obviously higher than that in wild-type lines, and slightly increased in *Wl-AG11–1* lines (Fig. 8b). *ARF2* and *TCP3* were down-regulated in *Sl-TeAGL11–1* lines, and *TCP3* was significantly decreased in *Wl-TeAGL11–1* lines. Expression levels of *GRF1*, *GRF2*, *GRF5* and *TCP20* had no significant changes, compared with those in wild type (Fig. 8b, Table S10).

## Discussion

### Evolutionary conservation and diversity of marigold *AGAMOUS*-like genes

In flowering plants, numerous MADS-box genes are important regulators for plant growth and development. The *AG* subfamily MADS-box genes are involved in regulating floral meristem and fruit development, and specifying reproductive organ identity in many species. Previous studies demonstrated that *AG* subfamily genes originated from several paraphyletic lineages in flowering plants, most of which probably arose from multiple whole-genome duplication events (WGDs) in flowering plants during long-term evolutionary process [9–12]. *AG* and *AGL11* lineages might arise from the first WGDs [10, 11]. The *AG* lineage underwent second WGDs in lower eudicots, resulting in the generation of two sub-clades, namely, *euAG* and *PLE* [9, 10]. Recently, duplication events of *PLE* lineages observed in Arabidopsis [23] and tomato [39] made their function different from that of *euAG*. In marigold, TeAG1, TeAG2, TeAGL11–1, and TeAGL11–2 were grouped into AG subfamily proteins. These four proteins contained highly conservative AG motif I and AG motif II in the C-terminal regions (Fig. 1) [10, 40]. According to our phylogenetic analysis, TeAG1 and TeAG2 belonged to euAG lineages, TeAGL11–1 and TeAGL11–2 were classified into AGL11 lineages, whereas no PLE lineage proteins were found in marigold transcriptome data (Fig. 2). Dreni and Kater’s proposal suggests that PLE lineage proteins might lost in Asteraceae family, which might be related to dry, indehiscent seed in Asteraceae [9].

### Specific expressions of marigold *AGAMOUS*-like genes in inner two whorls of floral organs and ovules

In marigold, we found that the expressions of four *AG* subfamily genes were highly tissue-specific. The expression patterns of *TeAG1* and *TeAG2* (C class genes) in floral organs were consistent with those reported in Gerbera [35]. These two genes were preferentially expressed in reproductive organ and ovules (Fig. 3a, b, Fig. S1a, b), indicating that *TeAG1* and *TeAG2* might play an important role in regulating the reproductive organ development and specifying ovary identity. Furthermore, *TeAG1* and *TeAG2* were slightly expressed in sepals and petals, suggesting *TeAG1* might be involved in regulating four-whorl floral organ developments. Unlike *TeAG2*, *TeAG1* was highly expressed in pistils and ovaries, indicating their functional differentiation. Similar to *TeAG1* and *TeAG2* expression pattern, *TeAGL11–1* and *TeAGL11–2* (D class genes) were highly expressed in pistils and ovaries (Fig. 3a, b, S1a-d), suggesting that both C class and D class genes in marigold might be involved in regulating pistil and ovary development in the evolutionary process. The expression patterns of *TeAGL11–1* and *TeAGL11–2* were clearly different from each other. *TeAGL11–1* was highly expressed in disk flower organs including stamens, sepals, and ovaries, while *TeAGL11–2* was highly expressed in pistils and ovules (Fig. 3a, b, S1c, d). Combining the above finding with the fact of low amino acid sequence identity between TeAGL11–1 and TeAGL11–2 proteins, we speculated that these two genes might have various functions in regulating flower development. The expression patterns of *TeAGL11–1* and *TeAGL11–2* differed from those of *FBP7*, *FBP11,* or *STK* whose expressions were limited to seed and ovule [41, 42]. Thus, the specific expression of D class genes in marigold suggested some extra functions of *TeAGL11–1* or *TeAGL11–2* in addition to regulating seed development.

### Conservative role of *TeAG1* in specifying stamen and carpel identities

Compared to previous studies in other plants, the similar phenotypic changes of flower organs and seed development were observed in *35S:TeAG1* transgenic lines. The ectopic expression of *TeAG1* in Arabidopsis resulted in homeotic conversion of sepals into pistilliod structures and that of petals into stamenoid structures (Figs. 5e, g, 6e, f, h, l, Table 2), which was consistent with the results of ectopic expression of C class genes in Chrysanthemum [43] and Carnation (*Dianthus caryphyllus*) [44]. For example, ectopic expression of *CDM37* (C class gene) in Chrysanthemum resulted in the conversion of petals into antheroid structure, and that of sepals into pistilloid tissues [43]. Based on ABCDE model, ectopic expression of functional class C orthologue would suppress the A class homeotic genes expression in the first and second whorls, leading to the transformation of sepals and petals into carpels and stamens, respectively. The analysis of the expression pattern of endogenous genes *AP1*, *AP3*, *PI*, *AG,* and *STK* revealed that *TeAG1* developed activities to repress A class genes (Fig. 6v). Based on the specific expression of *TeAG1* and the noticeable phenotypic alteration of transgenic plants, we speculated that *TeAG1* might regulate the stamen and carpel developments.

### Function of *TeAGL11–1* in seed and petal development

The phenotypical changes caused by *TeAGL11–1* overexpression in Arabidopsis suggested that D class genes might have conservative and divergent function. The almost seedless phenotype in *35S:TeAGL11–1* lines was similar to that in other plant species in which D class gene expression were suppressed, suggesting that the function of class D genes in transgenic Arabidopsis is partially inhibited. In Arabidopsis, the triple *stk/shp1/shp2* abolished the development of ovule and seed [24]. In Petunia, simultaneous down-regulation of *FBP7* and *BP11* formed the seedless phenotype [18, 20, 41]. In addition to model plants, similar phenotypes resulting from down-regulation the D class genes were also observed in other species [2, 3]. The down-regulation expression of endogenous gene *STK* also supported this phenotypic change (Fig. 7q), suggesting that ectopic expression *TeAGL11–1* in Arabidopsis led to co-suppression phenotype. Co-suppression phenomenon was observed in other ectopic expression lines of MADS-box genes. For example, overexpression *HAM45as* and *HAM59* (C class) genes from Sunflower in tobacco downregulated the expression level of endogenous C class genes, further resulting in the conversion of stamen into petals [45]. In addition, the curled petals in *35S:TeAGL11–1* lines were observed, but this phenotype was different from those resulting from overexpression of D class genes in Arabidopsis and tomato where the sepals were transformed into carpeloid organs bearing ovules [42] or a fleshy organ [3]. On one hand, the different phenotypes might be attributed to the functional difference between *TeAGL11–1* and *STK* (Arabidopsis) or *Sl-AGL11*(tomato, *Solanum lycopersicum*). On the other hand, different gene regulatory networks in marigold and Arabidopsis might lead to their difference in heterologous transformation phenotype. The decrease in *PI* transcript level might explain the petal curling (Fig. 7q).

### Functional conservation and diversity of *TeAG1* and *TeAGL11–1* genes

The early flowering phenotype was observed in transgenic plants containing *35S:TeAG1* and *35S:TeAGL11–1* constructs (Figs. 5c, l, 7c, p, Table 2), which was consistent to the phenotype of transgenic Arabidopsis with the overexpression of *AG* or *STK* [42, 46]. In addition, overexpression *TeAG1* and *TeAGL11–1* in Arabidopsis led to rosette leaf curling (Figs. 5b, 7b, l, Table 2). Similar phenotype was also observed in transgenic plants in which C or D class genes were ectopically expressed [47, 48]. Remarkably, in Arabidopsis, ectopic expressions of *TeAG1* and *TeAGL11–1* resulted in short siliques, low seed setting rate, and sepal retainment at the bottom of mature siliques. This result was consistent with the finding reported in Cymbidium [49]. The expression analysis of endogenous gene *STK* suggested that short siliques with low seed setting rate might be correlated with the increased *STK* expression level in *35S:TeAG1* lines, while the short siliques with low seed setting rate might be associated with the decreased *STK* transcript level in *TeAGL11–1* transgenic lines. In general, these results supported our prediction that ectopic expression of C and D class genes might have similar functions in regulating the floral development in Arabidopsis [50]. In addition, the divergent function between *TeAG1* and *TeAGL11–1* were also observed. Compared with phenotypic changes of *35S*:*TeAG1* transgenic plants, the overexpression of *TeAGL11–1* caused the petals to curl inwards, but it could not induce homologous alteration of floral organs (Fig. 7e, f, h, n, Table 2).

Previous studies indicated that the early flowering and curled leaves caused by overexpression of MADS-box genes might be usually associated with expression changes of upstream or downstream genes [51–54]. In this study, the transcript levels of endogenous genes regulating flowering time and curled leaves in *35S:TeAG1*, *35S:TeAGL11–1* lines and wild-type plants were analyzed. The results indicated that the transcript levels of *AP1*, *FT*, *AG,* and *SEP3* were up-regulated in *35S:TeAG1* and *35S:TeAGL11–1* lines. Previous report on soybean (*Glycine max*) indicated that the early flowering was related to the up-regulated transcript levels of *AP1*, *FT*, and *SEP3* [55]*.* Taken together, the increased expression levels of *AP1*, *FT*, *AG,* and *SEP3* might promote the formation of flowers and early flowering in transgenic plants (Fig. 8a, b). The analysis of gene expression and phenotypic changes revealed that the leaf curling in *35S:TeAG1* transgenic lines might be correlated with the transcript changes of *GRF2* and *TCP18* (Fig. 8a), which was consistent with results that *TCP* [56] and *GRF* [57] could regulate leaf development. In addition, the down-regulation of *ARF2* and *TCP3* and up-regulation of *TCP18* might result in the curled leaves in *35S:TeAGL11–1* lines (Fig. 8b). The expression of *LFY* was evidently increased in transgenic lines with higher expression level of *TeAG1*, suggesting the expression levels of *LFY* might be related to the transgene expression.

## Conclusions

This study reveals that *TeAG1* and *TeAGL11–1* regulate the development of floral organs, seeds and vegetative tissues, and that *TeAG2* and *TeAGL11–2* might lose their ability to regulate the floral organ and ovules developments, or they need bind with other MADS-box genes to regulate the flower development and ovules formation. Our results expand the understanding of the development of stamens, pistils and ovules in Asteraceae family, and provide technical support for the subsequent creation of horticultural traits. However, further situ hybridization analysis and homologous transformation phenotyping experiments are still needed to explore the accurate expression regions and potential functional mechanism of these four genes.

## Methods

### Plant materials and growth conditions

An inbred line M525B-1 of marigold was derived from 10 generations of continuous self-crossing of M525B which is a male fertile type plant from the two-type (male sterile/male fertile) line M525AB isolated by He [58] in our lab. M525B-1 had one whorl of ray florets in the periphery of the capitulum. They were grown in the natural conditions in fall 2016 at Huazhong Agricultural University, Wuhan, Hubei Province, China (lat. 30°28′36.5“ N, long, 114°21’59.4” E).

The seeds from *Arabidopsis thaliana* accession Columbia (Col-0) plants used for the functional analysis were first sterilized and cultured on the agar containing Skoog (MS) and 1/2× Murashige at 4 °C for 2 days. After 10 days, the seedlings were transplanted to growth chamber under long-day conditions (16 h light/8 h dark) at 22/20 °C day/night temperature with 60% relative humidity until genetic transformation.

### Total RNA extraction, isolation and bioinformatics analysis of *AG* and *AGL11* genes from marigold

Various vegetative tissues, floral buds in four developmental stages, and floral tissues from marigold were collected and quickly frozen in liquid nitrogen as described by Ai et al. [33]. There were four different sizes of flower buds: FB1: 0–1 mm in diameter, the ray floret primordium and the outermost disk floret primordium were formed; FB2: 2–3 mm in diameter, the sepals and petals of the ray florets and the outmost disk florets were developed; FB3: 4–5 mm in diameter, the stamens of the outmost disk florets were developed; and FB4: 6–7 mm in diameter, the pistils of the ray florets and the outmost disk florets were developed. Total RNA of each sample was isolated by PLANTpure kit (Aidlab, Beijing, China) according to the manufacturer’s protocol. The quality and quantity of RNA were tested by a Nano-Drop 2000 Spectrophotometer (Thermo Fisher Scientific, Wilmington, DE). The first-strand cDNA was synthesized by using PrimeScriptTM RT reagent Kit with gDNA Eraser (Takara, Dalian, China). Based on the transcriptome sequence (accession number SRP066084) [33], four *AG* subfamily genes were selected and named *TeAG1* (comp38613_c0, comp38613_c1), *TeAG2* (comp68705_c0), *TeAGL11–1* (comp199520_c0), and *AGL11–2* (comp50841_c0). The full length of *TeAG1*, *TeAG2*, *TeAGL11–1*, *TeAGL11–2* were cloned with the specific primers *TeAG1*-*Full*-F/R, *TeAG2*-*Full*-F/R, T*eAGL11–1*-*Full*-F/R, and *TeAGL11–2*-*Full*-F/R, respectively (Supplementary Table S1). The PCR amplified products were purified and cloned into *pMD18-T* vector (Takara, Dalian, China). Positive clones (3–5 replicates) were confirmed by sequencing in the Sangon company in Shanghai.

Multiple sequence alignment of TeAG1, TeAG2, TeAGL11–1, TeAGL11–2 proteins with other known C/D class genes was performed by using the DNAMAN (v.6.0) software (https://www.lynnon.com). To analyze the phylogenetic relationships of C/D class genes, a number of *AG* and *AGL11* genes were downloaded from the National Center for Biotechnology Information (NCBI) (http://www.ncbi.nlm.nih.gov). Full-length amino-acid sequences were first aligned using the default settings in MUSCLE implemented in MEGA (v. 6.0), and then adjusted manually with the reference alignment provided by Zahn et al. [59]. Phylogenetic tree was constructed by MEGA (v. 6.0) software by using the neighbor-joining (NJ) method under 1000 bootstrap replicates.

### Quantitative real-time PCR for expression analysis

Expression levels of the C/D class genes in different tissues (roots, tender stems, and fresh leaves), floral tissues (sepals, petals, and pistils of ray and disk florets, stamens of disk florets, receptacles, bracts, and ovaries), and different sizes of flower buds in marigold were analyzed by quantitative real-time PCR (qRT-PCR). Gene-specific primers (Supplementary Table S1) for qRT-PCR were designed within the non-conservative C-terminal region by the Primer Premier 5.0. QRT-PCR experiments were performed, as described in previous study [33]. The expression levels of these four genes per sample were repeated three times. In these experiments, the reference gene *beta*-*actin* was used for normalization and the relative expression levels were calculated using the 2^–ΔΔCt^ method. The data were analyzed using the TBtools software and normalized using row scale.

### Subcelluar localization of AG and AGL11 proteins from marigold

The coding sequences of *TeAG1*, *TeAG2*, *TeAGL11–1*, *TeAGL11–2* with removed stop codon were amplified and introduced into a pYellow vector under the control of the *35S CaMV* promoter to generate fusion vectors *35S:YFP-TeAG1*, *35S:YFP-TeAG2*, *35S:YFP-TeAGL11–1*, *35S:YFP-TeAGL11–2.* The primers were listed in supplementary Table S1. The YFP signal could be observed in both cytoplasm and nucleus when transfected with *35S: YFP* vector. The *35S:YFP* empty vector was used as a negative control, and the *35S:RFP*-N7 vector including the N7 nuclear targeting signal was used as a nucleus control. The control vectors and recombinant vectors were transformed into agrobacterium tumefacien strain *GV3101*, respectively. Then, the recombinant vectors and the nucleus control *35S:RFP*-N7 or the pYellow-*YFP* empty and nucleus control *35S:RFP*-N7 in tumefacien strain *GV3101* were simultaneously injected into tobacco (*Nicotiana benthamiana*) leaves, separately [60]. After incubation at 25 °C for 48 h, the YFP fluorescence signal and RED fluorescence signal in tobacco leaves were detected by confocal microscopy (Leica, TCS SP2, Wetzlar, Germany).

### Yeast two-hybrid assay

The cDNA of *TeAG1*, *TeAG2*, *TeAGL11–1*, and *TeAGL11–2* were amplified using primers with specific restriction sites (Table S1). The PCR fragments were recombined with the plasmid pGBKT7 and pGADT7 (Clontech, Palo Alto, CA, USA), respectively. The PGADT7 and PGBKT7 recombinant vectors were co-transformed into strain *AH109* by the LiAc/DNA/PEG method following the the Frozen-EZ Yeast Transformation II Kit protocols (Zymo Research Corp, Irvine, CA, USA). The transformants were selected on selection medium lacking leucine (Leu) and tryptophan (Trp). The autoactivation and toxicity of BD clones were tested by co-transforming with empty AD plasmid. Simultaneous transfer of empty AD and empty BD vectors or pGBKT7–53 and pGADT7-T vectors into *AH109* was set as the negative control or positive control. The positive yeast cells were verified by PCR with AD-F−/R and BD-F−/R (Supplementary Table S1). Then positive yeast cells were further tested by spotting assays on X-a-gal-supplemented medium without Leu, Trp, histidine (His), and adenine (Ade). The result was observed after incubation of plates at 30 °C for 3–5 days. In this study, the positive signal of interaction ability was scored if any direction interaction caused yeast to grow on the selection plate.

### Vectors construction and ectopic expression in Arabidopsis

The coding regions of *TeAG1*, *TeAG2*, *TeAGL11–1* and *TeAGL11–2* were amplified using specific primers with restriction sites (Supplementary Table S1), and then were cloned into *p2300* vector under the control of the *CaMV 35S* promoter. The obtained fusion vectors were named *35S:TeAG1*, *35S:TeAG2*, *35S:TeAGL11–1,* and *35S:TeAGL11–2.* These fusion vectors were introduced into *Escherichia coli DH5a,* and tested by PCR. These fusion vectors were then transformed into *Agrobacterium tumefaciens* strain *GV3101*. These resultant constructs were transformed into wild-type Arabidopsis ecotype Columbia plants by using the floral dip method [61]. Transformants were selected on a medium containing 50 μg ml^− 1^ *kanamycin,* and were further verified by PCR and semi-quantitative RT-PCR (semi-RT-PCR) analysis with the primers *35S*-F, *35S-TeAG1*-R, *35S-TeAG2*-R, *35S-TeAGL11–1*-R, *35S-TeAGL11–2*-R, qRT-*TeAG1*-F/R, qRT-*TeAG2*-F/R, RT-*TeAG11–1*-F/R, RT-*TeAGL11*–2-F/R, and *AtEF1α*-F/R (Supplementary Table S1). Phenotype changes of T1 and T2 transgenic lines were analyzed.

### Scanning electron microscopy

The blooming flowers of *35S:TeAG1* and wild-type plants were collected and fixed overnight in 2.5% (v/v) glutaraldehyde at 4 °C. Then the flowers were dehydrated every 15 min with an ethanol series (30–100%). Ethanol was replaced with isoamyl acetate/ethanol (1/1) and isoamyl acetate for every 10 min. The dried samples were critical point dried (CPD 020; Balzers Union, http://www.bal-tec. com/), sputter-coated with gold (NanotechSEMPrep II sputtercoater, NanotechLtd., Prestwick, UK), and fixed on the specimen stubs by double-sided tape. The samples were examined and photographed using a LEO 435VP scanning electron microscope (LEO Electron Microscopy Ltd., http://www.smt.zeiss.com/).

### Expression analysis of endogenous genes in transgenic plants

To further analyze the mechanism of floral organs and seed phenotype changes in *35S:TeAG1* and *35S:TeAGL11–1* transgenic plants, the expression levels of *AP1*, *AP3*, *PI*, *AG* and *AGL11* of Arabidopsis were detected by qRT-PCR. Total RNA of blooming flowers from *35S:TeAG1*, *35S:TeAGL11–1* transgenic plants and wild-type plants was isolated by using PLANTpure (Aidlab, Beijing, China), and then reverse-transcribed by using PrimeScript™ RT reagent Kit with gDNA Eraser (Takara, Dalian, China). Arabidopsis *EF1*α (*AtEF1α*, AT5G60390) was used as housekeeping gene. In addition, the transcript levels of some endogenous genes related to flowering time (*FT*, *SOC1*, *LFY*, *AP1*, *SEP3*, *AG*) [54] and leaf development (*GRF1*, *GRF2*, *GRF5*, *TCP3*, *TCP18*, *TCP20*, and *ARF2*) were analyzed [62], when the transgenic and wild-type seedlings were 10 days old. All the samples of 10-day-old seedlings were collected at the same time under light conditions. The primers were listed in Supplementary Table S1.

## Supplementary information

**Additional file 1: Table S1.** Sequence of primers.

**Additional file 2: Table S2.** Amino acid sequence alignment of C class proteins.

**Additional file 3: Table S3.** Amino acid sequence alignment of D class proteins.

**Additional file 4: Fig. S1.** Expression levels of *TeAG1, TeAG2, TeAGL11–1* and *TeAGL11–2* in different tissues and organs. Rt: root; Sm: stems; Le: leaf; FB1-FB4: flower buds were 0-1 mm, 2-3 mm, 4–5 mm and 6-7 mm in diameter, respectively; Re: receptacle; Br: bract; RS: sepal of ray floret; RP: petal of ray floret; RPi: pistil of ray floret; Se: sepal of disk floret; Pe: petal of disk floret; St: stamen of disk floret; Pi: pistil of disk floret; Ov: ovary.

**Additional file 5: Fig. S2.** Interactions of TeAG and TeAGL11 proteins of marigold by yeast two-hybrid assays. (a) Assession of Self-activation and autoaction of AD and BD constructs. (b)Ten-fold serial dilutions from 10^− 1^ to 10^− 4^ of each culture were spotted on the selected SD -Leu/−Trp/−His/−Ade plates with X-α-gal.

**Additional file 6: Fig. S3.** Expression of *TeAG1, TeAG2, TeAGL11–1* and *TeAGL11–2* in seedlings of T1 transgenic lines by semi-quantitative RT-PCR. (a-1) *35S:TeAG1* transgenic lines. (b-1) *35S:TeAG2* transgenic lines. (c-1) *35S:TeAG11–1* transgenic lines. (d-1) *35S:TeAG11–2* transgenic lines. WT: wild type line; SL: strong phenotypic line; WL: weak phenotypic line; L: transgenic line. (a-2, b-2, c-2, d-2), the constitutive gene is Arabidopsis keeping-house gene *AtEF1α. (DOCX 654 kb)*

**Additional file 7: Table S4.** Statistics for seed setting rate between control and *Sl-TeAG1* transgenic lines.

**Additional file 8: Table S5.** Statistics for seed setting rate between control and *Sl-TeAGL11–1* transgenic lines.

**Additional file 9: Table S6.** Raw data of C_T_ value in qRT-PCR for expression levels of *TeAG1*, *TeAG2*, *TeAGL11–1*, and *TeAGL11–2* in different tissues and organs of marigold.

**Additional file 10: Table S7.** Raw data of C_T_ value in qRT-PCR for expression levels of *AP1*, *AP3*, *PI*, *AG*, and *STK* in flowers from *35S:TeAG1* transgenic lines and wild-type Arabidopsis.

**Additional file 11: Table S8.** Raw data of C_T_ value in qRT-PCR for expression levels of *AP1*, *AP3*, *PI*, *AG*, and *STK* in flowers from *35S:TeAGL11–1* transgenic lines and wild-type Arabidopsis.

**Additional file 12: Table S9.** Raw data of C_T_ value in qRT-PCR for expression levels of *AP1*, *AP3*, *PI*, *AG*, and *STK* in seedlings of *35S:TeAG1* transgenic lines and wild-type Arabidopsis.

**Additional file 13: Table S10.** Raw data of C_T_ value in qRT-PCR for expression levels of *AP1*, *AP3*, *PI*, *AG*, and *STK* in seedlings of *35S:TeAGL11–1* transgenic lines and wild-type Arabidopsis.

## Data Availability

The sequences information of AGAMOUS-like proteins was uploaded to the NCBI (TeAG1: MT452648, TeAG2: MT452649, TeAGL11–1: MT394168, and TeAGL11–2, MT394169).

## Abbreviations

AP1: APETALA1
AP3: APETALA1
SEP3: SEPALLATA3
PI: PISTILLATA
TCP: TEOSINTEBRANCHED 1, CYCLOIDEA, PROLIFERATING FACTOR
GRF: GROWTH-REGULATING FACTOR
ARF2: AUXIN RESPONSE REGULATOR 2
FT: FLOWERING LOCUS T
SOC1: SUPPRESSOR OF OVEREXPRESSION CO 1
LFY: LEAFY
AG: AGAMOUS
WGDs: whole-genome duplication events
AGAL11: AGAMOUS-LIKE11
PLE: PLENA
FBP7: FLORAL BINDING PROTEIN 7
FBP11: FLORAL BINDING PROTEIN 11
STK: SEEDSTICK
SHP1: SHATTERPROOT1
SHP2: SHATTERPROOT1
YFP: yellow fluorescent protein
RFP: red fluorescent protein
Leu: leucine
Trp: tryptophan
His: histidine
Ade: adenine
RT-qPCR: quantiative real-time PCR
Semi-PCR: semi-quantitative-PCR
