# Supplementary material for: Identification, characterization and functional analysis of AGAMOUS subfamily genes associated with floral organs and seed development in Marigold (Tagetes erecta)
Source: BMC Plant Biol. 2020 Sep 23;20:439. doi: 10.1186/s12870-020-02644-5 (PMC7510299; doi:10.1186/s12870-020-02644-5)
Supplement: Supplementary file 1 — Additional file 1: Table S1. Sequence of primers. [file 12870_2020_2644_MOESM1_ESM.docx]

**Table S1.** Sequence of primers

| Primers | Sequences (5’-3’) | remarks |
| --- | --- | --- |
| *TeAG1-Full*-F | GGAAGGTCCTCAAAAAGCAAG | Full-length gene cloning |
| *TeAG1-Full*-R | AATCATCACATCCGGCTTCGA | Full-length gene cloning |
| *TeAG2-Full*-F | TTCCTGTTCAGATTTGTGACAAACC | Full-length gene cloning |
| *TeAG2-Full*-R | CCTCGATCAAATTCTTTATGCCCAT | Full-length gene cloning |
| *TeAGL11-1-Full-*F | AAGCTGGAGGAGATATGGGGAGA | Full-length gene cloning |
| *TeAGL11-1-Full*-R | ATTGTCGTGGTCATCCTCGCT | Full-length gene cloning |
| *TeAGL11-2-Full*-F | TCTTCTAAGGCAAAGTCAAACACGG | Full-length gene cloning |
| *TeAGL11-2-Full*-R | GTGGCTAAAACGCCTCAAAATAACC | QRT-PCR |
| qRT-*TeAG1* -F | GCCTGGAGGTTCTGCTTATG | QRT-PCR, Semi-RT-PCR |
| qRT-*TeAG1*-R | TCCTGGCAAGAGTAGTCGTTAT | QRT-PCR R, Semi-RT-PCR |
| qRT-*TeAG2* -F | TTGATGCCTGGAAGTTCTGATT | QRT-PCR R |
| qRT-*TeAG2*-R | GTTATTCGGTTGAAGATCGTTGAC | QRT-PCR |
| qRT-*TeAGL11-1 -*F | CATACCATGCAGCTACCAGAA | QRT-PCR |
| qRT-*TeAGL11-1* -R | ATAAAGGTGCGTAGGTTGATCC | QRT-PCR |
| qRT-*TeAGL11-2* -F | CGCACAACAACACAGTCCT | QRT-PCR, Semi-quantitative RT-PCR |
| qRT-*TeAGL11-2* -R | GGCACTTCTAGCAAGGTAAGC | QRT-PCR, Semi-quantitative RT-PCR |
| RT-*TeAG2* -F | GCAAGTTAGAGAAAGGTATTAGCAG | Semi-quantitative RT-PCR |
| RT -*TeAG2*-R | AATGGTTGATGAGGCGGCAC | Semi-quantitative RT-PCR |
| RT -*TeAGL11-1 -*F | CTTATGGGTGAAGGATTAGGCTG | Semi-quantitative RT-PCR |
| RT -*TeAGL11-1* -R | GCTTGTGTGGAATGGGGGTA | Semi-quantitative RT-PCR |
| WSJ=RT-ACT-F | GGGAAATGAATGCCAAAGCCAAG | QRT-PCR |
| WSJ=RT-ACT-R | AAGACTTCACAACCACTCTCCAACT | QRT-PCR |
| YFP -*TeAG1* -F | **GGATCC**ATGGCAAATTCTGATCAGGCTAA | Subcelluar localization |
| YFP -*TeAG1*-R | **GTCGAC**CACCAACTGGAGAGGGGTTTGG | Subcelluar localization |
| YFP -*TeAG2* -F | **GAATTC**ATGTCTTTTCCTAATGAGTCAGGGG | Subcelluar localization |
| YFP -*TeAG2*-R | **GTCGAC**CACTAACTGGAGAGGTGTTTGATCT | Subcelluar localization |
| YFP -*TeAGL11-1 -*F | **GGATCC**ATGGGGAGAGGAAGAATTGAGAT | Subcelluar localization |
| YFP -*TeAGL11-1* -R | **GTCGAC**CCCAAGATGAAGAAAAGGCTTG | Subcelluar localization |
| YFP -*TeAGL11-2* -F | **GAATTC**ATGGGTAGAGGAAGGATTGAGATCA | Subcelluar localization |
| YFP -*TeAGL11-2* -R | **GGATCC**CCAGATGTGAAGAGACTTGTTGGG | Subcelluar localization |
| AD/BD -*TeAG1* -F | **CATATG**ATGGCAAATTCTGATCAGGCTAAT | Yeast two-hybrid |
| AD/BD -*TeAG1*-R | **GGATCC**TCACACCAACTGGAGAGGGGTTTGG | Yeast two-hybrid |
| AD/BD-*TeAG2* -F | **CATATG**ATGTCTTTTCCTAATGAGTCAG | Yeast two-hybrid |
| AD/BD -*TeAG2*-R | **GAATTC**TTACACTAACTGGAGAGGTG | Yeast two-hybrid |
| AD/BD -*TeAGL11-1 -*F | **GAATTC**ATGGGGAGAGGAAGAATTGAGATA | Yeast two-hybrid |
| AD/BD -*TeAGL11-1* -R | **GGATCC**TCACCCAAGATGAAGAAAAGGCTT | Yeast two-hybrid |
| AD/BD-*TeAGL11-2* -F | **GAATTC**ATGGGTAGAGGAAGGATTGAGATCA | Yeast two-hybrid |
| AD/BD -*TeAGL11-2* -R | **GGATCC**TCACCAGATGTGAAGAGACTTGTTG | Yeast two-hybrid |
| AD-F | CTATTCGATGATGAAGATACCCCACCAAACCC | Yeast two-hybrid |
| AD-R | GTGAACTTGCGGGGTTTTTCAGTATCTACGATT | Yeast two-hybrid |
| BD-F | TCATCGGAAGAGAGTAG | Yeast two-hybrid |
| BD-R | GAGTCACTTTAAAATTTGTAT | Yeast two-hybrid |
| *35S-TeAG1*-F | **GGTACC**ATGGCAAATTCTGATCAGGCTAATG | Ectopic expression vector |
| *35S-TeAG1*-R | **TCTAGA**TCACACCAACTGGAGAGGGGTTTGG | Ectopic expression vector |
| *35S-TeAG2*-F | **GGTACC**ATGTCTTTTCCTAATGAGTCAG | Ectopic expression vector |
| *35S-TeAG2*-R | **TCTAGA**TTACACTAACTGGAGAGGTG | Ectopic expression vector |
| *35S-TeAG11-1*-F | **GGTACC**ATGGGGAGAGGAAGAATTGAGATA | Ectopic expression vector |
| *35S-TeAG11-1*-R | **TCTAGA**TCACCCAAGATGAAGAAAAGGCTTG | Ectopic expression vector |
| *35S-TeAG11-2*-F | **GGTACC**ATGGGTAGAGGAAGGATTGAGATC | Ectopic expression vector |
| *35S-TeAG11-2*-R | **TCTAGA**TCACCAGATGTGAAGAGACTTGTTG | Ectopic expression vector |
| *35S*-F | ACGCACAATCCCACTATCCTTC | General primer of 35S promoter |
| *FT*-qF | TATCCCTGCTACAACTGGAACAACC | QRT-PCR |
| *FT*-qR | GCCTGCCAAGCTGTCGAAACAATAT | QRT-PCR |
| *SOC1*-qF | TGAAAGCGAAGTTTGGTCAAATAAGA | QRT-PCR |
| *SOC1*-qR | TCTTGAAGAACAAGGTAACCCAATGA | QRT-PCR |
| *LFY*-qF | TACTCTCCGCCGCTGGTGATTC | QRT-PCR |
| *LFY*-qR | ACTTCCTCCTCCGCCGTTATTCC | QRT-PCR |
| *SEP3*-qF | GTATCAGGGGCAACAAGATGGAAT | QRT-PCR |
| *SEP3*-qR | AAAGAGAGGGATTGATTAAGTGAGAAAGA | QRT-PCR |
| *AG*-qF | CTACGAGCAGCTTATGCCACCA | QRT-PCR |
| *AG*-qR | GAGTAATGGTGATTGTTAGGTTGC | QRT-PCR |
| *ARF2*-qF | GCTTGTGCTGGTCCGCTTGT | QRT-PCR |
| *ARF2*-qR | TGTTCTGCCGCCTGGTTCGT | QRT-PCR |
| *TCP20*-qF | TGATGATGCTGCTTCTGCTGTTGT | QRT-PCR |
| *TCP20*-qR | GCTGGTTCTGATTCTGACTCTTCTTGT | QRT-PCR |
| *TCP18*-qF | ACGACAATCATCATCCATCACTTCACT | QRT-PCR |
| *TCP18*-qR | TTGCTGTGCCTGTCCGTTCTG | QRT-PCR |
| *TCP3*-qF | TTGCTTCTCCGTCGTCGTTGC | QRT-PCR |
| *TCP3*-qR | ACTGCTGATGATGATGATGATGATGGT | QRT-PCR |
| *GRF5*-qF | ACTTCTCAGTTCAATGTCTTAGCCTCT | QRT-PCR |
| *GRF5*-qR | CGGTTGTTGGTGATGATGGTATGGA | QRT-PCR |
| *GRF2*-qF | GAACGACAACTAGAGCGGAAGAAGA | QRT-PCR |
| *GRF2*-qR | CTTGACGGCGAGAATCAGAGGAA | QRT-PCR |
| *GRF1*-qF | CTCGGCAAGCAGCACCATCATT | QRT-PCR |
| *GRF1*-qR | ACAGAGAAGGAGCAGTAGCAGAAGTAT | QRT-PCR |
| *AtEF1α-*F | GCAAGATGGATGCCACTACCC | QRT-PCR, Semi-quantitative RT-PCR |
| *AtEF1α-*R | AGTGGGAGACGAAGGGGCT | QRT-PCR, Semi-quantitative RT-PCR |

Note: bold font is the sequence of enzyme cutting site.
