# Supplementary material for: Identification, characterization and functional analysis of AGAMOUS subfamily genes associated with floral organs and seed development in Marigold (Tagetes erecta)
Source: BMC Plant Biol. 2020 Sep 23;20:439. doi: 10.1186/s12870-020-02644-5 (PMC7510299; doi:10.1186/s12870-020-02644-5)
Supplement: Supplementary file 2 — Additional file 2: Table S2. Amino acid sequence alignment of C class proteins. [file 12870_2020_2644_MOESM2_ESM.docx]

**Table S2.** Amino acid sequence alignment of C class proteins.

|  | TeAG1 | TeAG2 | HAM45 | HAM59 | AG |
| --- | --- | --- | --- | --- | --- |
| TeAG1 | 100% | 84.90% | 85.77% | 84.08% | 62.55% |
| TeAG2 | 84.90% | 100% | 85.89% | 97.57% | 70.28% |

Note: HAM45 (*Helianthus annuus*, accession number: AO18228), HAM59 (*Helianthus annuus*, accession number: AAO18229), AG (*Arabidopsis thaliana*, accession number: CAA37642 AG).
