# Supplementary material for: Identification, characterization and functional analysis of AGAMOUS subfamily genes associated with floral organs and seed development in Marigold (Tagetes erecta)
Source: BMC Plant Biol. 2020 Sep 23;20:439. doi: 10.1186/s12870-020-02644-5 (PMC7510299; doi:10.1186/s12870-020-02644-5)
Supplement: Supplementary file 3 — Additional file 3: Table S3. Amino acid sequence alignment of D class proteins. [file 12870_2020_2644_MOESM3_ESM.docx]

**Table S3****.** Amino acid Sequence alignment of D class proteins.

|  | TeAGL11-1 | AGL11-2 | HaAGL11-1 | HaAGL11-2 | FBP11 | FBP7 |
| --- | --- | --- | --- | --- | --- | --- |
| TeAGL11-1 | 100% | 55.17% | 70.76% | 59.15% | 66.37% | 61.23% |
| TeAGL11-2 | 55.17% | 100% | 62.72% | 65.38% | 57.39% | 57.02% |

Note: HaAGL11-1 (*Helianthus annuus*, accession number: XP 021983447), HaAGL11-2 (*Helianthus annuus*, accession number: XP 022038697), FBP7 (*Petunia hybrida*, accession number: CAA57311), FBP11 (*Petunia hybrida*, accession number: CAA57445).
