# Supplementary material for: Identification, characterization and functional analysis of AGAMOUS subfamily genes associated with floral organs and seed development in Marigold (Tagetes erecta)
Source: BMC Plant Biol. 2020 Sep 23;20:439. doi: 10.1186/s12870-020-02644-5 (PMC7510299; doi:10.1186/s12870-020-02644-5)
Supplement: Supplementary file 4 — Additional file 4: Fig. S1. Expression levels of TeAG1, TeAG2, TeAGL11–1 and TeAGL11–2 in different tissues and organs. Rt: root; Sm: stems; Le: leaf; FB1-FB4: flower buds were 0-1 mm, 2-3 mm, 4–5 mm and 6-7 mm in diameter, respectively; Re: receptacle; Br: bract; RS: sepal of ray floret; RP: petal of ray floret; RPi: pistil of ray floret; Se: sepal of disk floret; Pe: petal of disk floret; St: stamen of disk floret; Pi: pistil of disk floret; Ov: ovary. [file 12870_2020_2644_MOESM4_ESM.docx]

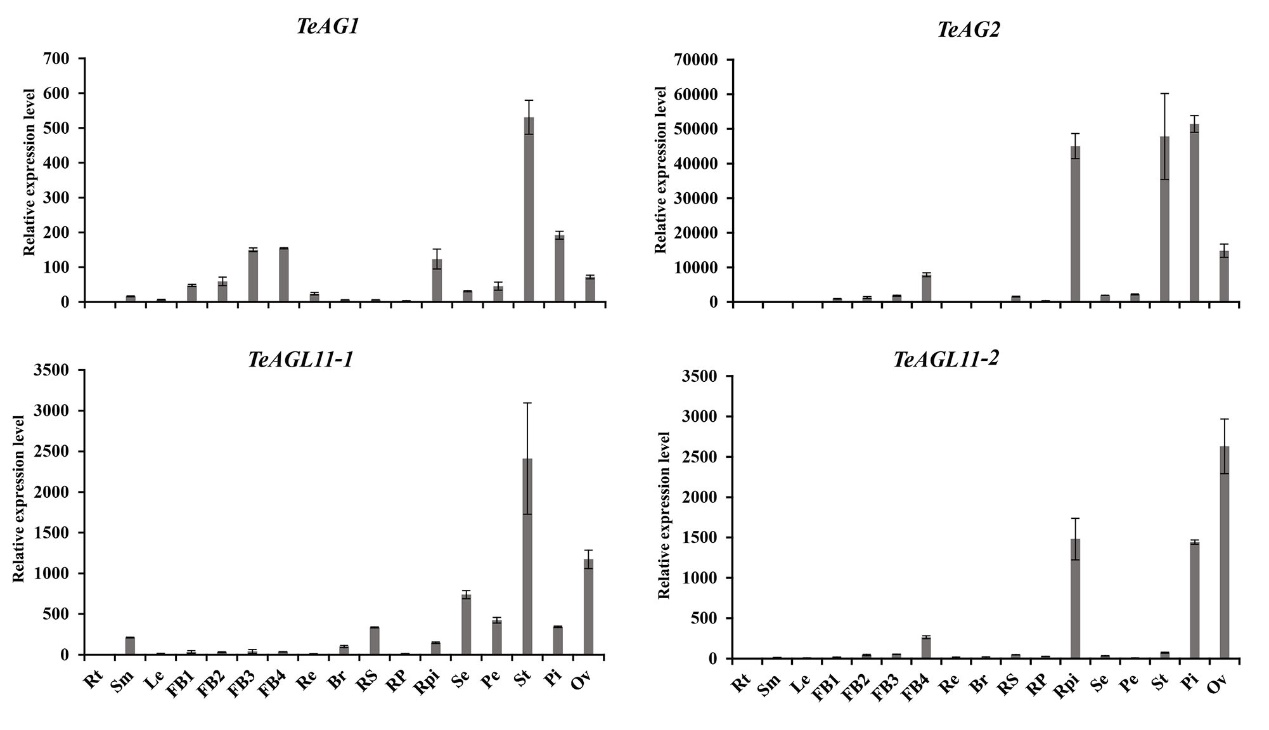


**Fig. S1 Expression levels of *TeAG1, TeAG2, TeAGL11-1* and *TeAGL11-2* in different tissues and organs.** Rt: root; Sm: stems; Le: leaf; FB1-FB4: flower buds were 0-1mm, 2-3mm, 4-5 mm and 6-7mm in diameter, respectively; Re: receptacle; Br: bract; RS: sepal of ray floret; RP: petal of ray floret; RPi: pistil of ray floret; Se: sepal of disk floret; Pe: petal of disk floret; St: stamen of disk floret; Pi: pistil of disk floret; Ov: ovary.
