# Supplementary material for: Identification, characterization and functional analysis of AGAMOUS subfamily genes associated with floral organs and seed development in Marigold (Tagetes erecta)
Source: BMC Plant Biol. 2020 Sep 23;20:439. doi: 10.1186/s12870-020-02644-5 (PMC7510299; doi:10.1186/s12870-020-02644-5)
Supplement: Supplementary file 5 — Additional file 5: Fig. S2. Interactions of TeAG and TeAGL11 proteins of marigold by yeast two-hybrid assays. (a) Assession of Self-activation and autoaction of AD and BD constructs. (b)Ten-fold serial dilutions from 10− 1 to 10− 4 of each culture were spotted on the selected SD -Leu/−Trp/−His/−Ade plates with X-α-gal. [file 12870_2020_2644_MOESM5_ESM.docx]

**a**


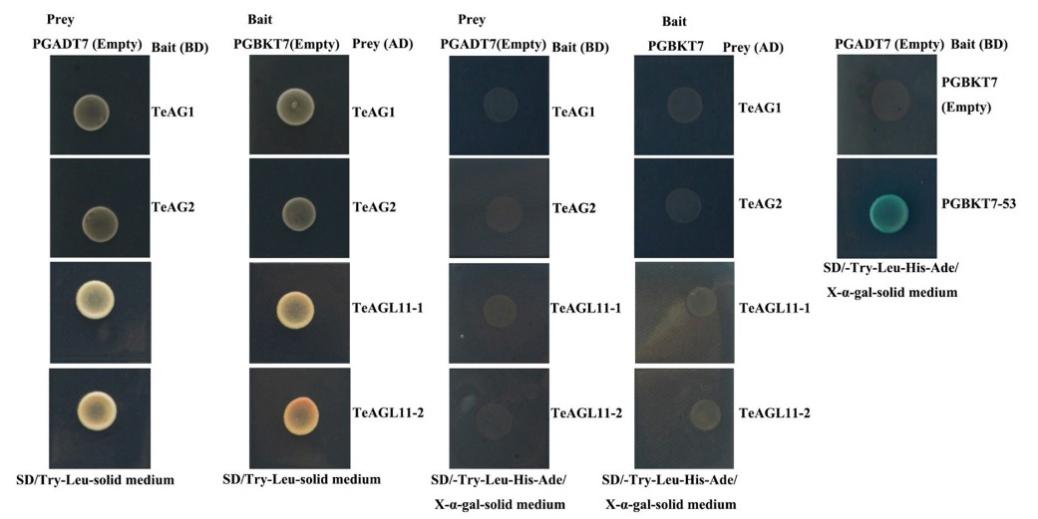


**b**


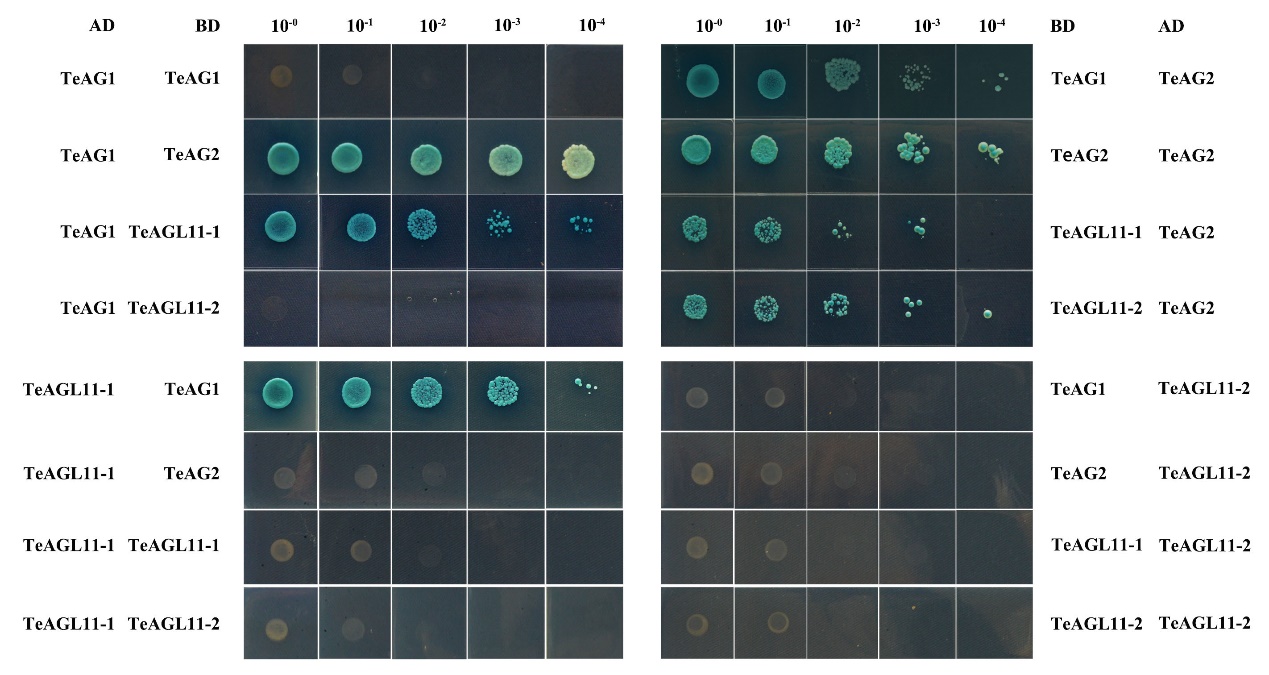


**Fig. S2. Interactions of TeAG and TeAGL11 proteins of marigold by yeast two-hybrid assays. (a)** Assession of Self-activation and autoaction of AD and BD constructs. **(b)**Ten-fold serial dilutions from 10^-1^ to 10^-4^ of each culture were spotted on the selected SD -Leu/-Trp/-His/-Ade plates with X-α-gal.
