# Supplementary material for: Identification, characterization and functional analysis of AGAMOUS subfamily genes associated with floral organs and seed development in Marigold (Tagetes erecta)
Source: BMC Plant Biol. 2020 Sep 23;20:439. doi: 10.1186/s12870-020-02644-5 (PMC7510299; doi:10.1186/s12870-020-02644-5)
Supplement: Supplementary file 6 — Additional file 6: Fig. S3. Expression of TeAG1, TeAG2, TeAGL11–1 and TeAGL11–2 in seedlings of T1 transgenic lines by semi-quantitative RT-PCR. (a-1) 35S:TeAG1 transgenic lines. (b-1) 35S:TeAG2 transgenic lines. (c-1) 35S:TeAG11–1 transgenic lines. (d-1) 35S:TeAG11–2 transgenic lines. WT: wild type line; SL: strong phenotypic line; WL: weak phenotypic line; L: transgenic line. (a-2, b-2, c-2, d-2), the constitutive gene is Arabidopsis keeping-house gene AtEF1α. (DOCX 654 kb) [file 12870_2020_2644_MOESM6_ESM.docx]

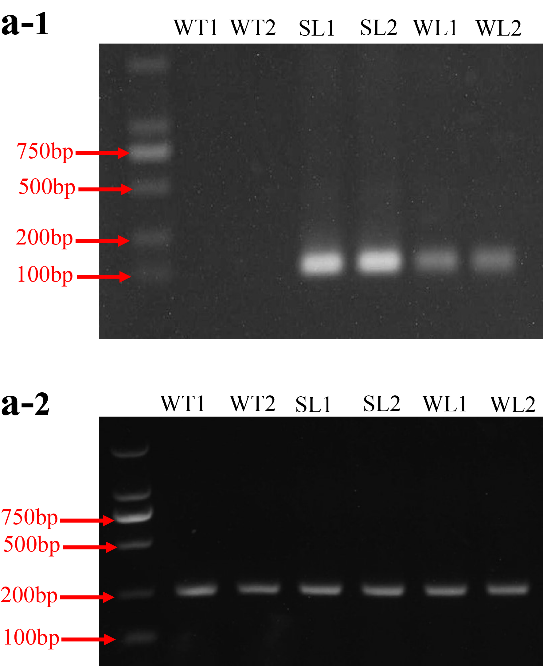

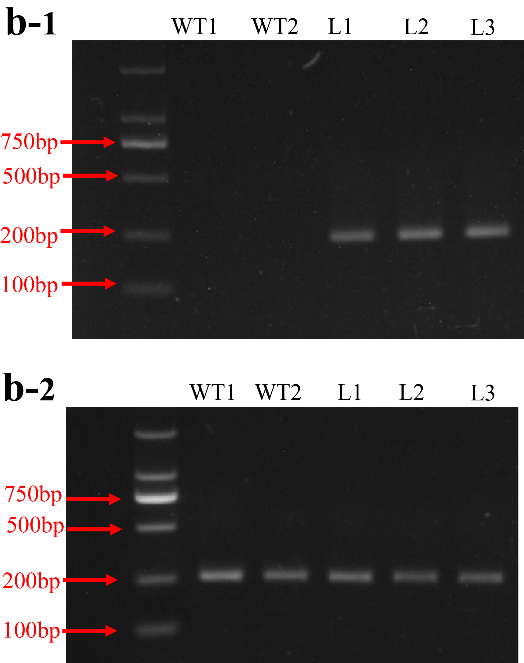


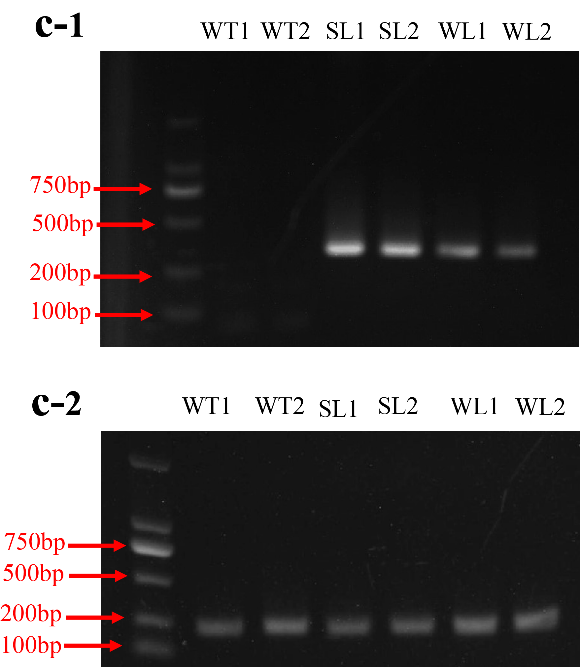

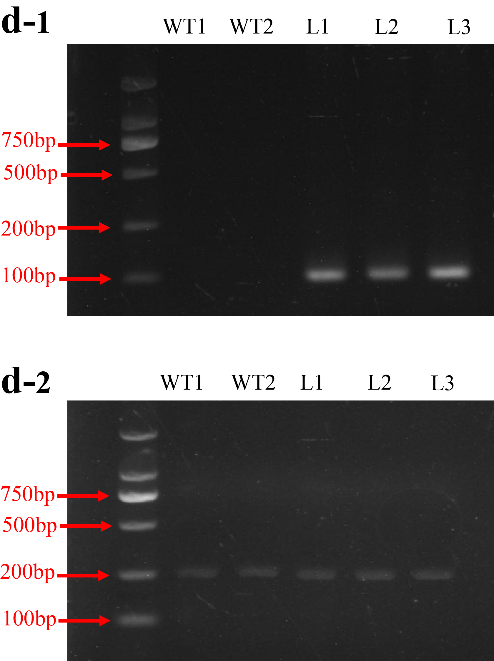


**Fig. S3 Expression of *TeAG1, TeAG2, TeAGL11-1* and *TeAGL11-2* in seedlings of T1 transgenic lines by semi-quantitative RT-PCR.** (**a-1**) *35S:TeAG1* transgenic lines. (**b-1**) *35S:TeAG2* transgenic lines. (**c-1**) *35S:TeAG11-1* transgenic lines. (**d-1**) *35S:TeAG11-2* transgenic lines. WT: wild type line; SL: strong phenotypic line; WL: weak phenotypic line; L: transgenic line. (**a-2, b-2, c-2, d-2**), the constitutive gene is Arabidopsis keeping-house gene *AtEF1α*
