# Supplementary material for: Identification, characterization and functional analysis of AGAMOUS subfamily genes associated with floral organs and seed development in Marigold (Tagetes erecta)
Source: BMC Plant Biol. 2020 Sep 23;20:439. doi: 10.1186/s12870-020-02644-5 (PMC7510299; doi:10.1186/s12870-020-02644-5)
Supplement: Supplementary file 8 — Additional file 8: Table S5. Statistics for seed setting rate between control and Sl-TeAGL11–1 transgenic lines. [file 12870_2020_2644_MOESM8_ESM.docx]

**Table S5.** Statistics for seed setting rate between control and *Sl-TeAGL11-1* transgenic lines.

|  | WT lines | *Sl-TeAGL11-1* transgenic lines |
| --- | --- | --- |
| Silique setting rate（%） | 100.00±0.00 | 15.57±4.43* |
| Number of seeds per silique (No.) | 49.42±3.85 | 7.14±2.54* |

* significant difference at P <0.05.
