# Supplementary material for: Identification, characterization and functional analysis of AGAMOUS subfamily genes associated with floral organs and seed development in Marigold (Tagetes erecta)
Source: BMC Plant Biol. 2020 Sep 23;20:439. doi: 10.1186/s12870-020-02644-5 (PMC7510299; doi:10.1186/s12870-020-02644-5)
Supplement: Supplementary file 9 — Additional file 9: Table S6. Raw data of CT value in qRT-PCR for expression levels of TeAG1, TeAG2, TeAGL11–1, and TeAGL11–2 in different tissues and organs of marigold. [file 12870_2020_2644_MOESM9_ESM.docx]

**Table S6**. Raw data of C_T_ value in qRT-PCR for expression levels of *TeAG1*, *TeAG2*, *TeAGL11-1*, and *TeAGL11-2* in different tissues and organs of marigold.

| Gene Name | Sample Name | Cт | | | | | | | | |
| --- | --- | --- | --- | --- | --- | --- | --- | --- | --- | --- |
|  |  | BR1 | | | BR2 | | | BR3 | | |
|  |  | TR1 | TR2 | TR3 | TR1 | TR2 | TR3 | TR1 | TR2 | TR3 |
| *WSJ=RT-ACT* | RT | 16.487 | 16.568 | 16.553 | 15.389 | 15.978 | 15.559 | 17.000 | 17.012 | 16.679 |
|  | Sm | 20.179 | 20.215 | 20.155 | 17.197 | 16.934 | 17.900 | 17.900 | 17.458 | 17.657 |
|  | Le | 18.980 | 19.140 | 19.283 | 17.368 | 17.000 | 17.100 | 17.264 | 17.018 | 16.929 |
|  | FB1 | 18.885 | 18.817 | 18.714 | 19.389 | 19.467 | 19.358 | 17.325 | 17.029 | 17.538 |
|  | FB2 | 18.993 | 19.556 | 19.168 | 19.239 | 19.769 | 18.521 | 18.163 | 18.763 | 18.638 |
|  | FB3 | 20.711 | 20.660 | 20.633 | 19.546 | 19.596 | 18.443 | 18.813 | 19.124 | 19.322 |
|  | FB4 | 20.412 | 20.510 | 20.490 | 18.463 | 18.369 | 18.487 | 18.926 | 18.500 | 18.729 |
|  | Re | 20.614 | 20.596 | 21.108 | 18.538 | 19.000 | 18.983 | 19.729 | 19.700 | 19.900 |
|  | Br | 19.159 | 18.969 | 18.971 | 17.346 | 17.900 | 17.772 | 17.999 | 18.111 | 18.000 |
|  | RS | 19.216 | 19.286 | 19.210 | 16.999 | 17.111 | 16.833 | 17.463 | 17.263 | 17.299 |
|  | RP | 17.824 | 17.815 | 17.780 | 15.997 | 16.008 | 15.900 | 16.527 | 16.890 | 16.468 |
|  | Rpi | 19.528 | 19.230 | 19.298 | 17.673 | 17.468 | 17.789 | 18.689 | 18.900 | 18.390 |
|  | Se | 20.099 | 20.127 | 20.111 | 18.264 | 18.000 | 18.086 | 19.000 | 18.892 | 18.800 |
|  | Pe | 19.284 | 19.515 | 19.332 | 16.953 | 17.300 | 17.136 | 17.683 | 17.579 | 17.264 |
|  | St | 19.501 | 19.605 | 19.585 | 17.026 | 17.522 | 17.363 | 17.511 | 17.235 | 17.339 |
|  | Pi | 18.959 | 18.982 | 18.987 | 16.126 | 16.353 | 16.003 | 17.000 | 16.999 | 16.793 |
|  | Ov | 17.894 | 18.254 | 18.064 | 16.683 | 17.000 | 16.889 | 16.996 | 16.828 | 17.156 |
| *TeAG1* | RT | 24.606 | 24.819 | 25.405 | 23.738 | 23.815 | 23.471 | 25.225 | 25.471 | 24.714 |
|  | Sm | 24.421 | 24.262 | 24.913 | 21.579 | 21.783 | 20.587 | 22.003 | 21.394 | 22.160 |
|  | Le | 24.716 | 24.677 | 24.963 | 22.604 | 22.499 | 22.842 | 22.338 | 22.503 | 22.241 |
|  | FB1 | 21.459 | 21.556 | 21.506 | 22.110 | 21.998 | 21.780 | 19.947 | 19.987 | 20.056 |
|  | FB2 | 21.531 | 21.579 | 21.902 | 21.768 | 21.694 | 21.651 | 20.668 | 20.488 | 20.461 |
|  | FB3 | 21.747 | 21.779 | 21.618 | 20.483 | 20.226 | 19.875 | 19.983 | 19.992 | 20.099 |
|  | FB4 | 21.448 | 21.478 | 21.400 | 19.263 | 19.348 | 19.582 | 19.693 | 19.426 | 19.839 |
|  | Re | 24.486 | 24.603 | 24.365 | 22.224 | 22.162 | 22.434 | 23.416 | 23.388 | 23.750 |
|  | Br | 24.749 | 24.602 | 24.857 | 22.929 | 23.109 | 23.026 | 23.557 | 23.565 | 23.785 |
|  | RS | 23.980 | 23.947 | 23.748 | 21.313 | 21.503 | 21.446 | 22.137 | 21.997 | 21.583 |
|  | RP | 24.116 | 24.106 | 24.334 | 22.513 | 22.347 | 22.134 | 22.728 | 22.607 | 22.754 |
|  | Rpi | 20.942 | 20.846 | 20.781 | 19.002 | 19.152 | 18.918 | 19.662 | 19.829 | 19.332 |
|  | Se | 23.492 | 23.347 | 23.487 | 21.293 | 21.250 | 21.307 | 22.093 | 22.225 | 22.132 |
|  | Pe | 21.997 | 21.892 | 21.509 | 19.763 | 19.743 | 19.761 | 20.384 | 20.538 | 20.846 |
|  | St | 18.530 | 18.873 | 18.989 | 16.400 | 16.654 | 16.645 | 16.544 | 16.346 | 16.276 |
|  | Pi | 19.507 | 19.666 | 19.683 | 16.559 | 17.287 | 16.897 | 17.688 | 17.534 | 17.162 |
|  | Ov | 20.031 | 20.190 | 20.412 | 18.928 | 18.770 | 19.112 | 19.094 | 18.750 | 18.957 |
| *TeAG2* | RT | 32.483 | 32.385 | 32.181 | 31.003 | 31.083 | 30.853 | 32.929 | 33.182 | 32.983 |
|  | Sm | 30.184 | 30.030 | 30.353 | 27.103 | 27.061 | 27.331 | 27.473 | 27.574 | 27.663 |
|  | Le | 31.537 | 31.722 | 31.625 | 29.222 | 29.307 | 29.609 | 29.192 | 29.006 | 28.850 |
|  | FB1 | 24.677 | 24.690 | 24.699 | 25.542 | 25.207 | 25.583 | 23.476 | 23.407 | 22.168 |
|  | FB2 | 24.648 | 24.960 | 24.827 | 24.751 | 24.723 | 24.915 | 23.543 | 23.657 | 23.530 |
|  | FB3 | 25.589 | 25.990 | 25.544 | 24.012 | 24.142 | 24.207 | 23.676 | 23.656 | 24.287 |
|  | FB4 | 23.194 | 23.312 | 23.662 | 21.188 | 21.295 | 20.981 | 21.607 | 21.290 | 21.721 |
|  | Re | 32.235 | 32.533 | 33.255 | 30.237 | 30.583 | 30.032 | 31.193 | 31.524 | 31.675 |
|  | Br | 29.234 | 29.360 | 29.527 | 27.260 | 27.312 | 27.438 | 28.192 | 28.327 | 27.940 |
|  | RS | 24.366 | 24.593 | 24.416 | 21.993 | 21.925 | 22.183 | 22.525 | 22.427 | 22.495 |
|  | RP | 25.091 | 24.972 | 25.098 | 22.972 | 23.149 | 23.354 | 23.673 | 23.628 | 23.750 |
|  | Rpi | 19.860 | 19.695 | 19.740 | 17.882 | 17.832 | 17.777 | 18.954 | 19.003 | 18.943 |
|  | Se | 24.969 | 24.963 | 25.005 | 22.628 | 22.955 | 23.250 | 23.729 | 23.828 | 23.704 |
|  | Pe | 24.113 | 24.174 | 24.014 | 21.699 | 21.615 | 21.746 | 22.392 | 22.029 | 22.177 |
|  | St | 19.483 | 19.453 | 19.353 | 17.480 | 17.483 | 18.545 | 17.624 | 17.538 | 17.678 |
|  | Pi | 19.146 | 19.042 | 19.277 | 16.193 | 16.075 | 16.341 | 17.099 | 17.080 | 16.953 |
|  | Ov | 19.965 | 19.977 | 20.336 | 18.583 | 18.498 | 18.589 | 18.992 | 18.989 | 18.991 |
| *TeAGL11-1* | RT | 32.102 | 32.399 | 32.497 | 30.783 | 30.003 | 29.389 | 32.473 | 32.375 | 32.232 |
|  | Sm | 27.724 | 27.702 | 27.729 | 24.847 | 24.787 | 24.829 | 25.103 | 25.129 | 25.315 |
|  | Le | 31.259 | 31.170 | 31.332 | 28.249 | 28.208 | 28.194 | 28.689 | 28.017 | 29.289 |
|  | FB1 | 29.382 | 29.461 | 28.814 | 28.847 | 28.750 | 28.834 | 28.095 | 28.000 | 27.903 |
|  | FB2 | 29.731 | 29.722 | 29.743 | 29.091 | 29.111 | 29.588 | 29.879 | 29.004 | 27.817 |
|  | FB3 | 31.381 | 31.181 | 31.264 | 29.056 | 29.167 | 29.187 | 28.182 | 28.312 | 28.348 |
|  | FB4 | 30.624 | 30.572 | 30.560 | 28.188 | 28.474 | 28.619 | 29.077 | 28.989 | 28.480 |
|  | Re | 32.449 | 32.785 | 32.618 | 30.617 | 30.283 | 30.545 | 31.727 | 31.429 | 31.412 |
|  | Br | 27.625 | 27.432 | 27.831 | 26.042 | 26.127 | 25.911 | 26.703 | 26.703 | 26.953 |
|  | RS | 26.036 | 26.152 | 26.089 | 23.633 | 23.732 | 24.012 | 24.193 | 24.088 | 24.270 |
|  | RP | 29.443 | 29.531 | 29.233 | 27.131 | 27.228 | 27.434 | 28.382 | 28.374 | 28.249 |
|  | Rpi | 27.485 | 27.360 | 27.556 | 25.710 | 25.679 | 25.649 | 26.598 | 26.478 | 26.650 |
|  | Se | 25.857 | 25.866 | 25.813 | 23.803 | 23.698 | 24.153 | 24.387 | 24.535 | 24.522 |
|  | Pe | 25.968 | 25.889 | 25.928 | 23.387 | 23.876 | 23.237 | 23.992 | 24.103 | 24.170 |
|  | St | 23.202 | 23.998 | 24.027 | 21.638 | 21.459 | 21.410 | 20.903 | 20.929 | 21.027 |
|  | Pi | 25.734 | 25.829 | 25.872 | 23.029 | 22.829 | 22.937 | 23.873 | 23.779 | 23.560 |
|  | Ov | 23.069 | 23.169 | 23.386 | 21.991 | 22.062 | 21.739 | 21.829 | 21.729 | 22.055 |
| *TeAGL11-2* | RT | 30.717 | 30.987 | 31.254 | 29.925 | 30.121 | 29.106 | 31.117 | 31.038 | 31.382 |
|  | Sm | 30.973 | 30.755 | 31.231 | 27.582 | 27.773 | 27.960 | 28.327 | 28.228 | 28.365 |
|  | Le | 30.975 | 31.113 | 30.828 | 28.127 | 28.078 | 28.271 | 28.587 | 28.427 | 28.517 |
|  | FB1 | 28.992 | 28.984 | 28.975 | 29.493 | 29.425 | 29.572 | 27.563 | 27.458 | 27.380 |
|  | FB2 | 28.043 | 28.077 | 28.302 | 28.711 | 28.010 | 27.783 | 27.029 | 26.974 | 27.020 |
|  | FB3 | 29.209 | 29.174 | 29.070 | 27.768 | 27.654 | 27.856 | 27.520 | 27.654 | 27.759 |
|  | FB4 | 26.775 | 26.726 | 26.885 | 24.586 | 24.676 | 24.434 | 24.996 | 24.736 | 25.109 |
|  | Re | 32.345 | 32.138 | 32.719 | 28.834 | 28.640 | 29.207 | 30.000 | 29.998 | 31.912 |
|  | Br | 30.890 | 30.607 | 30.760 | 27.685 | 27.837 | 27.330 | 28.827 | 28.826 | 29.012 |
|  | RS | 27.975 | 27.884 | 28.207 | 25.227 | 25.583 | 25.940 | 26.053 | 26.054 | 26.093 |
|  | RP | 27.444 | 27.308 | 27.579 | 25.463 | 25.000 | 25.625 | 26.119 | 26.273 | 26.134 |
|  | Rpi | 23.315 | 23.169 | 23.356 | 21.228 | 21.122 | 21.035 | 22.400 | 22.328 | 22.643 |
|  | Se | 29.249 | 29.383 | 29.491 | 27.120 | 27.031 | 27.617 | 28.184 | 28.388 | 27.817 |
|  | Pe | 30.964 | 30.532 | 31.094 | 27.928 | 28.000 | 28.352 | 28.773 | 28.736 | 28.786 |
|  | St | 27.766 | 27.735 | 27.641 | 25.316 | 25.427 | 25.674 | 25.428 | 25.330 | 25.177 |
|  | Pi | 22.754 | 22.727 | 22.818 | 19.773 | 19.798 | 20.279 | 20.727 | 20.800 | 20.492 |
|  | Ov | 20.979 | 20.989 | 21.058 | 19.787 | 19.274 | 19.658 | 20.019 | 20.028 | 20.147 |

BR: biological replicates; TR: technical replicates
