# Supplementary material for: Identification, characterization and functional analysis of AGAMOUS subfamily genes associated with floral organs and seed development in Marigold (Tagetes erecta)
Source: BMC Plant Biol. 2020 Sep 23;20:439. doi: 10.1186/s12870-020-02644-5 (PMC7510299; doi:10.1186/s12870-020-02644-5)
Supplement: Supplementary file 10 — Additional file 10: Table S7. Raw data of CT value in qRT-PCR for expression levels of AP1, AP3, PI, AG, and STK in flowers from 35S:TeAG1 transgenic lines and wild-type Arabidopsis. [file 12870_2020_2644_MOESM10_ESM.docx]

**Table S7**. Raw data of C_T_ value in qRT-PCR for expression levels of *AP1*, *AP3*, *PI*, *AG*, and *STK* in flowers from *35S:TeAG1* transgenic lines and wild-type Arabidopsis.

| Gene name | Sample name | CT | | |
| --- | --- | --- | --- | --- |
|  |  | TR1 | TR2 | TR3 |
| *EF1α* | WT1 | 19.93683434 | 19.87515259 | 19.89061546 |
|  | WT2 | 15.50687408 | 15.52041912 | 15.55343819 |
|  | WL1 | 16.55189133 | 16.47351646 | 16.56212425 |
|  | WL2 | 15.7780962 | 15.9230547 | 15.78122997 |
|  | SL1 | 16.66345215 | 16.66745186 | 16.63013649 |
|  | SL2 | 16.22754002 | 16.2791748 | 16.32107925 |
| *AP1* | WT1 | 25.96732521 | 25.68527985 | 25.92623329 |
|  | WT2 | 20.92840576 | 21.00156975 | 21.00261497 |
|  | WL1 | 22.06640244 | 22.06181908 | 22.16614342 |
|  | WL2 | 22.45164967 | 23.22833252 | 22.60554314 |
|  | SL1 | 23.74502563 | 23.92334557 | 23.87300873 |
|  | SL2 | 23.14780235 | 23.41715622 | 23.40114212 |
| *AP3* | WT1 | 26.62399864 | 26.54208755 | 26.54557991 |
|  | WT2 | 21.93296127 | 21.71854362 | 21.90745049 |
|  | WL1 | 22.60186577 | 22.60264397 | 22.58372879 |
|  | WL2 | 22.08313084 | 22.17281055 | 21.93765736 |
|  | SL1 | 22.71774578 | 22.61924458 | 22.72618389 |
|  | SL2 | 21.73878765 | 21.72505379 | 21.73970222 |
| *PI* | WT1 | 26.39736176 | 26.28805733 | 26.22442055 |
|  | WT2 | 21.92506981 | 21.96092796 | 21.93138313 |
|  | WL1 | 22.06151199 | 21.72536469 | 21.92551231 |
|  | WL2 | 20.77787685 | 20.999547 | 20.81225491 |
|  | SL1 | 21.4435091 | 21.36104298 | 21.45122433 |
|  | SL2 | 20.18561649 | 20.06632805 | 20.17673683 |
| *AG* | WT1 | 26.9703846 | 26.9660778 | 27.01109314 |
|  | WT2 | 22.54427719 | 22.59381866 | 22.91180229 |
|  | WL1 | 23.26416397 | 23.15019608 | 23.25585365 |
|  | WL2 | 22.33320332 | 22.23670387 | 22.23325157 |
|  | SL1 | 21.85403442 | 21.92111588 | 21.72239113 |
|  | SL2 | 21.81657982 | 22.0330677 | 21.90149689 |
| *STK* | WT1 | 26.84906197 | 26.74019241 | 26.9160881 |
|  | WT2 | 22.99061966 | 22.74019241 | 22.5160881 |
|  | WL1 | 22.6171875 | 22.57588005 | 22.59305 |
|  | WL2 | 21.5041399 | 21.40284538 | 21.20491219 |
|  | SL1 | 21.92874908 | 21.86308479 | 21.77154732 |
|  | SL2 | 20.9343586 | 20.95781517 | 21.15075302 |

BR: biological replicates; TR: technical replicates
