# Supplementary material for: Identification, characterization and functional analysis of AGAMOUS subfamily genes associated with floral organs and seed development in Marigold (Tagetes erecta)
Source: BMC Plant Biol. 2020 Sep 23;20:439. doi: 10.1186/s12870-020-02644-5 (PMC7510299; doi:10.1186/s12870-020-02644-5)
Supplement: Supplementary file 11 — Additional file 11: Table S8. Raw data of CT value in qRT-PCR for expression levels of AP1, AP3, PI, AG, and STK in flowers from 35S:TeAGL11–1 transgenic lines and wild-type Arabidopsis. [file 12870_2020_2644_MOESM11_ESM.docx]

**Table S8**. Raw data of C_T_ value in qRT-PCR for expression levels of *AP1*, *AP3*, *PI*, *AG*, and *STK* in flowers from *35S:TeAGL11-1* transgenic lines and wild-type Arabidopsis.

| Gene name | Sample name | CT | | |
| --- | --- | --- | --- | --- |
|  |  | TR1 | TR2 | TR3 |
| *EF1α* | WT1 | 16.62635803 | 16.29387283 | 16.90584755 |
|  | WT2 | 16.80256844 | 16.72210121 | 16.53613091 |
|  | WL1 | 17.16108894 | 16.78806496 | 16.88180542 |
|  | WL2 | 17.73801231 | 18.04297829 | 18.10258865 |
|  | SL1 | 18.33749580 | 18.49104500 | 18.35979462 |
|  | SL2 | 13.95845127 | 13.69816685 | 13.77558613 |
| *AP1* | WT1 | 22.69411278 | 22.44103813 | 22.90820503 |
|  | WT2 | 22.73032570 | 22.39322281 | 22.28307533 |
|  | WL1 | 22.50920486 | 22.30229759 | 22.40946007 |
|  | WL2 | 23.53490448 | 23.84236908 | 23.92388153 |
|  | SL1 | 24.40630722 | 24.46545982 | 24.45873642 |
|  | SL2 | 20.15310669 | 20.19806576 | 20.21630287 |
| *AP3* | WT1 | 22.18619347 | 22.08269310 | 22.25798225 |
|  | WT2 | 22.37794685 | 22.21275330 | 22.08069038 |
|  | WL1 | 22.98334312 | 22.84454536 | 22.97414207 |
|  | WL2 | 23.45950699 | 23.72994041 | 23.88002396 |
|  | SL1 | 24.28001213 | 24.42575645 | 24.08568382 |
|  | SL2 | 20.12276268 | 19.73318100 | 20.02779007 |
| *PI* | WT1 | 18.80903435 | 18.79787064 | 18.99296951 |
|  | WT2 | 19.91322899 | 19.77373505 | 19.25731277 |
|  | WL1 | 21.41229248 | 21.27294731 | 21.07213783 |
|  | WL2 | 22.39163399 | 22.23786163 | 22.31843758 |
|  | SL1 | 22.43388748 | 22.48480797 | 22.51456642 |
|  | SL2 | 19.06109810 | 19.24877167 | 19.44382668 |
| *AG* | WT1 | 21.38473129 | 21.30690575 | 21.64917755 |
|  | WT2 | 20.92438698 | 20.99438858 | 20.79947853 |
|  | WL1 | 22.24416733 | 21.47183609 | 21.60536003 |
|  | WL2 | 23.91821861 | 24.09463310 | 24.07325935 |
|  | SL1 | 23.32159996 | 23.92264366 | 23.84102631 |
|  | SL2 | 21.26076317 | 21.15826797 | 21.67217064 |
| *STK* | WT1 | 22.06632423 | 21.98277283 | 22.09097481 |
|  | WT2 | 22.62697220 | 22.80195808 | 22.68574715 |
|  | WL1 | 23.65071487 | 23.15180016 | 23.08054543 |
|  | WL2 | 24.68784523 | 24.79329300 | 25.06217384 |
|  | SL1 | 25.83758354 | 25.95071220 | 25.92275047 |
|  | SL2 | 21.47268295 | 21.69736671 | 21.83800697 |

BR: biological replicates; TR: technical replicates
