# Supplementary material for: Identification, characterization and functional analysis of AGAMOUS subfamily genes associated with floral organs and seed development in Marigold (Tagetes erecta)
Source: BMC Plant Biol. 2020 Sep 23;20:439. doi: 10.1186/s12870-020-02644-5 (PMC7510299; doi:10.1186/s12870-020-02644-5)
Supplement: Supplementary file 12 — Additional file 12: Table S9. Raw data of CT value in qRT-PCR for expression levels of AP1, AP3, PI, AG, and STK in seedlings of 35S:TeAG1 transgenic lines and wild-type Arabidopsis. [file 12870_2020_2644_MOESM12_ESM.docx]

**Table S9**. Raw data of C_T_ value in qRT-PCR for expression levels of *AP1*, *AP3*, *PI*, *AG*, and *STK* in seedlings of *35S:TeAG1* transgenic lines and wild-type Arabidopsis.

| Gene name | Sample name | CT | | |
| --- | --- | --- | --- | --- |
|  |  | TR1 | TR2 | TR3 |
| *EF1α* | WT1 | 16.95020485 | 16.97436523 | 16.8869133 |
|  | WT2 | 16.18917084 | 16.26794434 | 16.33434677 |
|  | WL1 | 16.3399353 | 16.36771584 | 16.49162102 |
|  | WL2 | 16.53837013 | 16.70647049 | 16.88624573 |
|  | SL1 | 17.20754623 | 17.14014053 | 17.09846306 |
|  | SL2 | 17.18904877 | 17.17809296 | 16.9838829 |
| *AP1* | WT1 | 31.30984306 | 31.36188316 | 31.16127777 |
|  | WT2 | 31.02842331 | 31.34910393 | 31.45839119 |
|  | WL1 | 27.25522614 | 27.27597046 | 27.37467384 |
|  | WL2 | 26.96438408 | 26.99619865 | 27.02616119 |
|  | SL1 | 28.21941376 | 28.07346916 | 28.29531097 |
|  | SL2 | 28.08789444 | 27.96805191 | 27.87438583 |
| *AG* | WT1 | 30.33809662 | 30.36173058 | 30.13723373 |
|  | WT2 | 30.30286407 | 30.19522476 | 30.40319633 |
|  | WL1 | 28.5288887 | 28.53803444 | 28.6630497 |
|  | WL2 | 28.53092766 | 28.59798431 | 28.66862297 |
|  | SL1 | 28.77871323 | 28.76580048 | 28.73974037 |
|  | SL2 | 28.61831284 | 28.64100647 | 28.56806946 |
| *FT* | WT1 | 29.80653572 | 29.6714344 | 29.73351288 |
|  | WT2 | 29.31897545 | 29.66153908 | 29.51346588 |
|  | WL1 | 23.314291 | 23.3194313 | 23.3365612 |
|  | WL2 | 23.29990578 | 23.34901619 | 23.39667892 |
|  | SL1 | 23.49542618 | 23.46979523 | 23.3721714 |
|  | SL2 | 23.53022575 | 23.35379219 | 23.30657768 |
| *SEP3* | WT1 | 27.27972221 | 27.29110909 | 27.54236603 |
|  | WT2 | 27.48061752 | 27.28244209 | 28.36200523 |
|  | WL1 | 21.52216721 | 21.30648041 | 21.90538979 |
|  | WL2 | 21.72076607 | 21.74531364 | 21.84505653 |
|  | SL1 | 22.36700821 | 22.23805618 | 22.13455009 |
|  | SL2 | 22.14897156 | 22.17959023 | 21.90909958 |
| *SOC1* | WT1 | 26.36946487 | 26.48456001 | 26.26580048 |
|  | WT2 | 26.40802383 | 26.41674995 | 26.53164291 |
|  | WL1 | 24.30205345 | 24.39346313 | 24.4264679 |
|  | WL2 | 24.9741745 | 24.97365761 | 25.00382614 |
|  | SL1 | 24.88531494 | 24.84995651 | 24.82143021 |
|  | SL2 | 25.63609314 | 25.4931469 | 25.63147545 |
| *LFY* | WT1 | 27.18830872 | 27.35886765 | 27.12103462 |
|  | WT2 | 25.97704697 | 26.42194939 | 26.67696762 |
|  | WL1 | 25.81624031 | 26.03172112 | 26.08750534 |
|  | WL2 | 25.77929115 | 25.83717346 | 25.90563202 |
|  | SL1 | 25.99988747 | 26.18422318 | 26.09740257 |
|  | SL2 | 25.88665771 | 25.87550354 | 25.85366249 |
| *ARF2* | WT1 | 23.8699131 | 23.833498 | 23.66644478 |
|  | WT2 | 22.354105 | 22.48508072 | 22.54269028 |
|  | WL1 | 22.1120472 | 22.17300987 | 22.28285217 |
|  | WL2 | 22.03541183 | 22.38669014 | 22.51420975 |
|  | SL1 | 22.72458649 | 22.680233 | 22.57831383 |
|  | SL2 | 22.93585014 | 23.03893852 | 22.84912682 |
| *TCP20* | WT1 | 25.51410103 | 25.28007507 | 25.88294029 |
|  | WT2 | 24.59313583 | 24.60461426 | 24.72587585 |
|  | WL1 | 24.55057526 | 24.69638824 | 24.92720604 |
|  | WL2 | 24.86371422 | 24.94605827 | 24.99843597 |
|  | SL1 | 25.64226532 | 25.63342285 | 25.23870277 |
|  | SL2 | 26.32476807 | 26.12188339 | 26.12077522 |
| *TCP3* | WT1 | 23.06189346 | 23.14100838 | 22.97405624 |
|  | WT2 | 22.23652649 | 22.34028435 | 22.20266151 |
|  | WL1 | 21.31179047 | 21.41903687 | 21.80945969 |
|  | WL2 | 22.35623169 | 22.38179398 | 22.51379967 |
|  | SL1 | 22.53063774 | 22.51424026 | 22.35603905 |
|  | SL2 | 23.09466553 | 23.0724144 | 23.02922821 |
| *TCP18* | WT1 | 30.372118 | 29.64249992 | 30.60809898 |
|  | WT2 | 28.86055374 | 29.00967789 | 28.97680283 |
|  | WL1 | 25.86633682 | 25.86617851 | 26.11237526 |
|  | WL2 | 26.61565971 | 26.73344803 | 26.77429008 |
|  | SL1 | 26.75263596 | 26.63272476 | 26.34939575 |
|  | SL2 | 27.19981194 | 27.3741169 | 27.18201828 |
| GRF1 | WT1 | 25.49418449 | 25.47517776 | 25.4564724 |
|  | WT2 | 25.25324059 | 25.33312798 | 25.35983658 |
|  | WL1 | 24.04904366 | 23.92288971 | 24.16648865 |
|  | WL2 | 24.35810852 | 24.44907379 | 24.49363518 |
|  | SL1 | 24.7249527 | 24.71307755 | 24.10792923 |
|  | SL2 | 24.98476601 | 24.86929512 | 24.78463364 |
| *GRF2* | WT1 | 27.18647766 | 27.32787704 | 27.12263489 |
|  | WT2 | 26.73653603 | 26.82750702 | 26.94472313 |
|  | WL1 | 24.88046455 | 24.96270752 | 25.10984039 |
|  | WL2 | 25.55833435 | 25.59925652 | 25.87253571 |
|  | SL1 | 25.38045311 | 25.42098522 | 25.56969643 |
|  | SL2 | 26.32793129 | 26.25083572 | 26.07915382 |
| *GRF5* | WT1 | 25.74841309 | 25.86113548 | 25.56806946 |
|  | WT2 | 25.31326294 | 25.39954376 | 25.4094162 |
|  | WL1 | 24.92338181 | 24.8224678 | 24.95870018 |
|  | WL2 | 25.4318943 | 25.49356651 | 25.58169174 |
|  | SL1 | 26.0793705 | 25.75728226 | 25.66196632 |
|  | SL2 | 26.62219429 | 26.10699081 | 26.04175377 |

BR: biological replicates; TR: technical replicates
