# Supplementary material for: Identification, characterization and functional analysis of AGAMOUS subfamily genes associated with floral organs and seed development in Marigold (Tagetes erecta)
Source: BMC Plant Biol. 2020 Sep 23;20:439. doi: 10.1186/s12870-020-02644-5 (PMC7510299; doi:10.1186/s12870-020-02644-5)
Supplement: Supplementary file 13 — Additional file 13: Table S10. Raw data of CT value in qRT-PCR for expression levels of AP1, AP3, PI, AG, and STK in seedlings of 35S:TeAGL11–1 transgenic lines and wild-type Arabidopsis. [file 12870_2020_2644_MOESM13_ESM.docx]

**Table S10**. Raw data of C_T_ value in qRT-PCR for expression levels of *AP1*, *AP3*, *PI*, *AG*, and *STK* in seedlings of *35S:TeAGL11-1* transgenic lines and wild-type Arabidopsis.

| Gene name | Sample name | CT | | |
| --- | --- | --- | --- | --- |
|  |  | TR1 | TR2 | TR3 |
| *EF1α* | WT1 | 16.52964973 | 16.48032188 | 16.47503281 |
|  | WT2 | 16.61013985 | 16.57934952 | 16.786726 |
|  | WL1 | 15.88666153 | 15.83097172 | 15.9369688 |
|  | WL2 | 17.00394821 | 16.89644241 | 17.08009529 |
|  | SL1 | 18.98749733 | 19.02353096 | 19.0341568 |
|  | SL2 | 17.94139481 | 17.96435165 | 17.97974586 |
| *AP1* | WT1 | 29.89038658 | 29.66268349 | 29.47571564 |
|  | WT2 | 28.98641396 | 29.00787926 | 29.25167084 |
|  | WL1 | 26.56885529 | 26.54825783 | 26.61838341 |
|  | WL2 | 29.15571404 | 29.52895546 | 29.3071785 |
|  | SL1 | 28.76476479 | 28.74649239 | 28.79574776 |
|  | SL2 | 27.70602417 | 27.66402626 | 27.69619751 |
| *AG* | WT1 | 21.28802299 | 21.4933548 | 21.21836281 |
|  | WT2 | 21.23195839 | 20.86163689 | 21.25007362 |
|  | WL1 | 16.96712875 | 16.88995457 | 16.87468815 |
|  | WL2 | 19.61936188 | 19.1759758 | 19.64675522 |
|  | SL1 | 19.48272705 | 19.03137779 | 19.23454094 |
|  | SL2 | 18.21205521 | 17.70945549 | 17.72134972 |
| *FT* | WT1 | 28.45009804 | 28.48520088 | 28.36889648 |
|  | WT2 | 28.35861588 | 28.2986927 | 28.32376099 |
|  | WL1 | 25.13597107 | 25.28215599 | 25.22170258 |
|  | WL2 | 25.89812851 | 25.81972122 | 25.6419735 |
|  | SL1 | 27.71676178 | 27.76652336 | 27.86870384 |
|  | SL2 | 26.14204788 | 26.2095356 | 26.2426033 |
| *SEP3* | WT1 | 28.01073265 | 27.97405052 | 28.06246185 |
|  | WT2 | 28.03777504 | 27.99201584 | 28.02422905 |
|  | WL1 | 23.56516647 | 23.38476753 | 23.59134865 |
|  | WL2 | 24.45117378 | 24.43176651 | 24.47876549 |
|  | SL1 | 25.6302681 | 25.66692924 | 25.64107132 |
|  | SL2 | 24.58963776 | 24.56731033 | 24.45779037 |
| *SOC1* | WT1 | 23.88361549 | 23.87413406 | 24.05015755 |
|  | WT2 | 24.20564651 | 24.25889778 | 24.20523834 |
|  | WL1 | 23.35709763 | 23.41864967 | 23.42797279 |
|  | WL2 | 24.44438362 | 24.13170433 | 25.16279793 |
|  | SL1 | 25.05215263 | 25.07129669 | 25.02465248 |
|  | SL2 | 24.67775345 | 24.7489624 | 24.70915222 |
| *LFY* | WT1 | 28.52446175 | 28.5473175 | 28.45370293 |
|  | WT2 | 27.89463997 | 28.46193504 | 28.23198128 |
|  | WL1 | 27.68849182 | 27.68737793 | 27.63750267 |
|  | WL2 | 28.39017296 | 28.25450325 | 28.52755165 |
|  | SL1 | 29.4023056 | 29.27732086 | 29.33338737 |
|  | SL2 | 28.52446175 | 28.5473175 | 28.45370293 |
| *ARF2* | WT1 | 21.53643417 | 21.50672722 | 21.76816177 |
|  | WT2 | 21.9437542 | 22.78029442 | 21.8960247 |
|  | WL1 | 22.89941025 | 22.82254219 | 22.8653965 |
|  | WL2 | 23.34507561 | 23.18232346 | 23.47727776 |
|  | SL1 | 25.79744148 | 25.83225632 | 25.69014168 |
|  | SL2 | 23.70631218 | 23.76365471 | 23.80730057 |
| *TCP20* | WT1 | 25.72230148 | 25.70964622 | 25.75234032 |
|  | WT2 | 25.8893261 | 25.90027428 | 25.87538528 |
|  | WL1 | 26.0601635 | 26.11190224 | 26.1031189 |
|  | WL2 | 26.73502159 | 26.97117805 | 26.70745277 |
|  | SL1 | 28.58166885 | 28.73738098 | 28.75143814 |
|  | SL2 | 27.50716972 | 27.44360733 | 27.34544754 |
| *TCP3* | WT1 | 21.72485161 | 21.73739624 | 21.74871445 |
|  | WT2 | 21.9427166 | 21.98969078 | 21.75786018 |
|  | WL1 | 22.13880157 | 22.09787178 | 22.16862869 |
|  | WL2 | 23.65992165 | 23.75489044 | 23.78431892 |
|  | SL1 | 24.662117 | 24.57336617 | 24.51185799 |
|  | SL2 | 23.53395271 | 23.50866699 | 23.5196743 |
| *TCP18* | WT1 | 30.08433533 | 29.3065834 | 29.8392334 |
|  | WT2 | 29.93563843 | 30.03466797 | 29.12574196 |
|  | WL1 | 28.12976456 | 28.72305584 | 27.65809536 |
|  | WL2 | 29.73298931 | 29.27922726 | 29.03027534 |
|  | SL1 | 30.2429142 | 30.32974815 | 30.04162216 |
|  | SL2 | 28.71795273 | 28.35434723 | 28.77485275 |
| GRF1 | WT1 | 25.04084587 | 25.17666245 | 25.19077873 |
|  | WT2 | 25.3823185 | 25.64560509 | 25.22561264 |
|  | WL1 | 24.13458443 | 24.18099213 | 24.17596817 |
|  | WL2 | 25.17271614 | 25.348423 | 25.08043671 |
|  | SL1 | 26.63016701 | 26.92336464 | 26.80539131 |
|  | SL2 | 25.98953629 | 25.86911392 | 25.98620415 |
| *GRF2* | WT1 | 26.23954201 | 26.21030426 | 26.26857567 |
|  | WT2 | 26.70254326 | 26.68061638 | 26.68399811 |
|  | WL1 | 25.14925957 | 25.11761189 | 25.20702076 |
|  | WL2 | 26.35616684 | 26.20471191 | 26.98204422 |
|  | SL1 | 29.18634033 | 28.97514153 | 29.06141472 |
|  | SL2 | 27.71459198 | 27.78372955 | 27.71961975 |
| *GRF5* | WT1 | 24.53470421 | 24.53457451 | 24.45170403 |
|  | WT2 | 24.43535805 | 24.30879784 | 24.75385284 |
|  | WL1 | 25.23251724 | 25.32509995 | 24.92215347 |
|  | WL2 | 25.41400337 | 25.08884811 | 25.35088158 |
|  | SL1 | 25.80412292 | 25.95193291 | 25.87033844 |
|  | SL2 | 25.3645401 | 25.3983078 | 25.31415367 |

BR: biological replicates; TR: technical replicates
